# Generation of a *NES*-mScarlet Red Fluorescent Reporter Human iPSC Line for Live Cell Imaging and Flow Cytometric Analysis and Sorting Using CRISPR-Cas9-Mediated Gene Editing

**DOI:** 10.3390/cells11020268

**Published:** 2022-01-13

**Authors:** Parivash Nouri, Anja Zimmer, Stefanie Brüggemann, Robin Friedrich, Ralf Kühn, Nilima Prakash

**Affiliations:** 1 Laboratory of Applied Genetics and Stem Cell Biology, Department Hamm 2, Hamm-Lippstadt University of Applied Sciences, 59063 Hamm, Germany; Parivash.Nouri@hshl.de (P.N.); Stefanie.Brueggemann@stud.hshl.de (S.B.); Robin.Friedrich@stud.hshl.de (R.F.); 2 Genome Engineering & Disease Models, Max Delbrück Center for Molecular Medicine in the Helmholtz Association (MDC), 13125 Berlin, Germany; Anja.Zimmer@mdc-berlin.de

**Keywords:** iPSC, NESTIN, reporter, CRISPR-Cas9, neural stem cells, midbrain dopamine neurons

## Abstract

Advances in the regenerative stem cell field have propelled the generation of tissue-specific cells in the culture dish for subsequent transplantation, drug screening purposes, or the elucidation of disease mechanisms. One major obstacle is the heterogeneity of these cultures, in which the tissue-specific cells of interest usually represent only a fraction of all generated cells. Direct identification of the cells of interest and the ability to specifically isolate these cells in vitro is, thus, highly desirable for these applications. The type VI intermediate filament protein NESTIN is widely used as a marker for neural stem/progenitor cells (NSCs/NPCs) in the developing and adult central and peripheral nervous systems. Applying CRISPR-Cas9 technology, we have introduced a red fluorescent reporter (mScarlet) into the *NESTIN* (*NES*) locus of a human induced pluripotent stem cell (hiPSC) line. We describe the generation and characterization of *NES*-mScarlet reporter hiPSCs and demonstrate that this line is an accurate reporter of NSCs/NPCs during their directed differentiation into human midbrain dopaminergic (mDA) neurons. Furthermore, *NES*-mScarlet hiPSCs can be used for direct identification during live cell imaging and for flow cytometric analysis and sorting of red fluorescent NSCs/NPCs in this paradigm.

## 1. Introduction

Human induced pluripotent stem cell (hiPSC) lines are derived from somatic cells by reprogramming and serve as an invaluable resource for studying human cell and tissue models in vitro. The hiPSCs enable research about the mechanisms of differentiation in early human development, the physiology of specific lineages in 2D cultures and of cellular interactions in 3D organoids [1,2]. Since hiPSC lines can be established from cohorts of patients and healthy donors, they are also excellent tools for deciphering disease mechanisms and the screening of new drugs in vitro [3,4]. Moreover, hiPSC-derived precursor and differentiated cells have the potential for cell replacement therapies to cure a variety of degenerative diseases affecting different organs, including the central nervous system (CNS) [5].

Parkinson’s Disease (PD), which is characterized by the inexorable degeneration of a particular type of dopamine (DA)-producing neurons in the human brain and is typically diagnosed only at an advanced stage of the disease, has received particular attention in the regenerative medicine field [6]. The loss of the midbrain dopaminergic (mDA) neurons, especially those of the substantia nigra pars compacta (SNc), leads to the typical motor symptoms of PD, including the inability to execute voluntary movements, tremor and rigidity [7]. Replacement of the degenerating mDA neurons by fetal-brain-derived mDA precursor cells, which are transplanted into the brains of PD patients, is capable of restituting the DA levels required for their appropriate motor function. This approach is considered a promising alternative to the current practice of pharmacotherapy or deep brain stimulation for the symptomatic treatment of this disease [6]. The restricted availability and ethical dispute of fetal-brain-derived cells has propelled the use of stem cell-derived mDA precursors in recent years, leading to several ongoing clinical trials [6]. The mDA precursors are generated from human pluripotent stem cells (PSCs) subjected to a strict sequence of defined media and soluble factor treatments in the culture dish, commonly seen as the directed differentiation of these cells [8]. However, high quality standards need to be applied for the derivation of these precursors from a PSC pool, including thorough assessment of their identity and purity [9]. The mDA lineage is characterized by the stage- and cell-type-specific expression of several transcription factors, such as forkhead box A2 (FOXA2), orthodenticle homeobox 2 (OTX2), engrailed homeobox 1 (EN1) and LIM homeobox transcription factor 1 alpha (LMX1A) in proliferating mDA neural stem/progenitor cells (NSCs/NPCs) and postmitotic precursors; whereas the paired-like homeodomain 3 (PITX3) transcription factor, the rate-limiting enzyme in DA synthesis tyrosine hydroxylase (TH) and the DA transporter (DAT/SLC6A3) are detected only in maturing mDA neurons [10,11,12]. Additional marker proteins are used to distinguish between the SNc DA neuron subset, preferentially expressing the aldehyde dehydrogenase 1 family member A1 (ALDH1A1) and dopamine receptor D2 (DRD2), and the ventral tegmental area (VTA) DA neuron subset, expressing the calcium (Ca^2+^) binding protein calbindin 1 (CALB1) and cholecystokinin (CCK) [10,12]. Furthermore, the absence of “classical” pluripotency markers, such as POU class 5 homeobox 1 (POU5F1/OCT4), SRY-box transcription factor 2 (SOX2) and Nanog homeobox (NANOG), and markers characteristic of other brain regions, including the forebrain gene forkhead box G1 (*FOXG1*), the diencephalic genes BarH-like homeobox 1 (*BARHL1*) and paired box 6 (*PAX6*) as well as the hindbrain gene homeobox A2 (*HOXA2*), is a necessary requirement for the use of the generated mDA precursors as cell replacements of the degenerating mDA neurons [9,13]. Such criteria ensure the low tumorigenicity and proper functional integration of the transplanted cells into the circuitry of the diseased brain. They also make the isolation and purification of the target cell type (e.g., mDA precursors) a highly desirable but critical endeavor in the regenerative stem cell field, given the still-remarkable heterogeneity of the generated cells in current PSC differentiation paradigms [6,9].

The type VI intermediate filament NESTIN is expressed in proliferating stem and progenitor cells from different tissues, including NSCs and NPCs of the CNS and peripheral nervous system, bone marrow mesenchymal and hematopoietic stem cells, hair follicle stem cells, developing myotomes, skeletal muscle satellite cells and pancreatic islet stem cells [14]. NESTIN expression is downregulated, whereas other cytoskeletal proteins, such as the class III beta-tubulin (TUBB3) in the neural lineage, are upregulated, as soon as these stem/progenitor cells differentiate into more committed cells [14,15,16]. NESTIN is, thus, used as an NSC/NPC-specific marker protein in the context of human neural differentiation. Indeed, green fluorescent reporter PSCs for this cell population have been generated using either the endogenous *NESTIN* (*NES*) locus or the regulatory regions located in the second intron of this gene to direct the expression of the transgene specific to NSCs/NPCs [17,18].

Here we report the generation and characterization of reporter hiPSC lines expressing the red fluorescent protein (RFP) mScarlet under the direct control of the *NES* locus. Using CRISPR-Cas9 technology, we targeted the C-terminus of NESTIN and inserted the mScarlet coding region, together with a self-cleaving 2A peptide sequence, immediately upstream of the stop codon. Through independent translation of the reporter and avoidance of a selection marker, perturbation of the targeted locus was minimal and ensured the faithful expression and function of both the NESTIN and mScarlet reporter proteins. Using a modified protocol for the directed differentiation into mDA neurons, we show that the new *NES*-mScarlet reporter hiPSC line is particularly valuable for the direct observation of NESTIN-positive human NSCs/NPCs in live cell imaging and flow cytometric analyses, including downstream applications upon cell sorting. The red color reporter is of particular utility when combined with probes requiring additional green fluorescence, such as indicators for Ca^2+^ imaging. The *NES*-mScarlet reporter hiPSC line could be useful for the generation of other tissue and cell types under the appropriate conditions and might support clinical applications for the screening of new drugs and cell replacement therapy beyond mere research.

## 2. Materials and Methods

### 2.1. hiPSC Cell Culture for Gene Targeting

Human XM001 iPS cells (https://hpscreg.eu (accessed on 11 January 2021), HMGU-001-A) [19] were derived from fibroblasts of a healthy female and were obtained from Heiko Lickert (Helmholtz Center Munich, Munich, Germany). XM001 hiPSCs were grown in Essential 8 Flex Medium (E8; Thermo Fisher Scientific, Waltham, MA, USA) on Vitronectin-coated (5 µg/mL) dishes and passaged every 2 to 3 days using DPBS (w/o Ca, Mg, Thermo Fisher Scientific/0.5 mM EDTA or Accutase (#A1110501; Thermo Fisher Scientific) for dissociation, followed by overnight incubation in E8 medium with ROCK inhibitor (Y27632, 10 µM/mL; Tebu-Bio, Offenbach, Germany). Subclones were frozen in Bambanker freezing medium (Nippon Genetics Europe, Düren, Germany) or E8/10% DMSO and stored in liquid nitrogen. The study was conducted according to the guidelines of the Declaration of Helsinki and approved by the Ethics Committee of the Ärztekammer Westfalen-Lippe and Westfälische Wilhelms Universität Münster (protocol code 2019-426-f-S on 17 September 2019).

### 2.2. Targeting of the Human NESTIN Locus

XM001 hiPSCs were transfected with a plasmid for the expression of Cas9, RFP ([20], Addgene ID 86987), a cloned sgRNA targeting the end of *NES* exon 4 (CTGGTCCTCAGGGGAGGACT), a plasmid for the expression of BCL-XL to reduce apoptosis [21] and the *NES*-targeting vector at equal ratios using Lipofectamine 3000 (Invitrogen, Carlsbad, CA, USA). The targeting vector (plasmid backbone pMA, manufactured by Thermo Fisher Scientific) comprised 1.5 kb of sequences located upstream and downstream of the *NES* stop codon with the in-frame insertion of a P2A peptide, preceded by a Furin cleavage site (RAKR) [22], and followed by the mScarlet coding sequence [23] upstream of the stop codon (see Appendix A). Transfected RFP^+^ cells were enriched by fluorescence-activated cell sorting, replated, expanded and subjected to a second cycle of transfection and cell sorting to increase the overall targeting efficiency. Sorted cells were plated at low density and single-cell-derived colonies were isolated after 2 to 3 weeks, expanded, frozen and used for the isolation of genomic DNA.

### 2.3. PCR Genotyping

**Figure 1:**
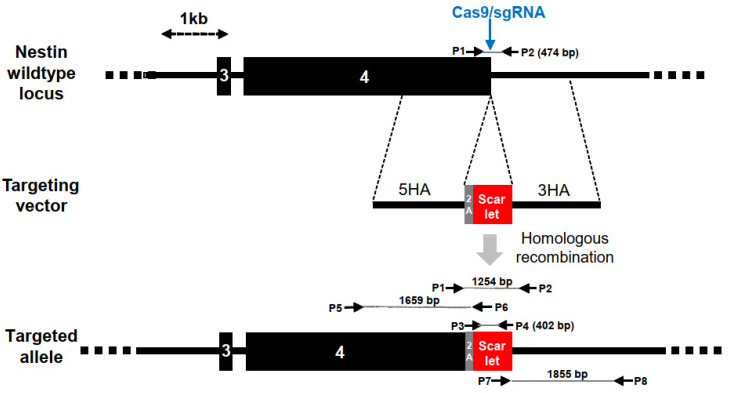
Insertion of mScarlet into the *NES* locus of human iPS cells (hiPSCs). The wildtype *NES* locus was cut with Cas9 and sgRNA spanning the stop codon at the end of exon 4. The targeted double-strand break could be repaired by homologous recombination with a targeting vector that included the in-frame insertion of a 2A peptide and the *mScarlet* coding sequence, flanked by 5′ and 3′ homology regions (5HA, 3HA). Upon the activation of the genomic locus, mScarlet was co-translated with NESTIN via the 2A peptide. The position of PCR primers, product sizes and a size scale are indicated.

Genotyping was performed by PCR on genomic DNA from transfected hiPSC clones using primer pairs P1/2, P3/4, P5/6 or P7/8 (Figure 1, Table 1) and Q5 (New England Biolabs, Ipswich, MA, USA) or Herculase II (Agilent, Santa Clara, CA, USA) DNA Polymerase at 63–65 °C annealing temperature. PCR reactions were analyzed by agarose gel electrophoresis. For Sanger sequencing, PCR bands were isolated using the GeneJET Gel Extraction Kit (Thermo Fisher Scientific), sequenced by LGC Genomics GmbH (Berlin, Germany) and analyzed using the CLC Main workbench software (Qiagen Digital Insights, Aarhus, Denmark).

### 2.4. Karyotype Analysis

hiPSCs were karyotyped with genomic DNA for SNP analysis using the Illumina OMNI-EXPRESS-8v1.6 Chip (Illumina, San Diego, CA, USA) (coverage of 958,497 markers spanning the human genome) and results were read with an Illumina iSCAN array scanner. Results were analyzed using Karyostudio 1.3 software, based on the information of the GRCh36/hg18 dataset. This method detects genomic gains and losses, copy number variants (CNVs), duplications/deletions, unbalanced translocations, aneuploidies, copy neutral aberrations and loss of heterozygosity/absence of heterozygosity.

### 2.5. Off-Target Site Prediction and Analysis

The sgRNA (CTGGTCCTCAGGGGAGGACT) on-target site and off-target sites were predicted using the CRISPOR webtool [24]. The off-target sites were analyzed as described in Figure S3.

### 2.6. Directed Differentiation of hiPSCs into mDA Neurons

Wildtype (*wt*) XM001 and *NES*-mScarlet hiPSCs (passage number 28) were maintained on hESC-qualified Matrigel (#354277; Corning, Amsterdam, The Netherlands)-coated plates in StemMACS™ iPS-Brew medium (#130-104-368; Miltenyi Biotec, Bergisch Gladbach, Germany). The pluripotency of hiPSC lines (quality control (QC) No. 1) was confirmed by quantitative RT-PCR (RT-qPCR) and ICC for pluripotency marker genes (Table 2) and proteins (Human Embryonic Stem Cell Marker Panel#ab238602; Abcam, Boston, MA, USA). Directed differentiation of the hiPSCs into mDA neurons was conducted in a monolayer culture using a modified protocol from [13,25,26], as follows. The hiPSCs were dissociated in 0.5 mM EDTA/DPBS (w/o Ca, Mg) and plated onto Matrigel (#356234; Corning) and 0.5 µg/cm^2^ of human recombinant laminin 111 (#LN111; BioLamina, Sundbyberg, Sweden)-coated tissue culture plastic at a density of 0.5–1.5 × 10^4^ cells/cm^2^ in N2 medium (1:1 mixture of DMEM:F12 (#21331020; Thermo Fisher Scientific) and Neurobasal (#21103049; Thermo Fisher Scientific), 1% N-2 supplement (#17502001; Thermo Fisher Scientific), 1% GlutaMAX (#35050038; Thermo Fisher Scientific) and 0.2% penicillin/streptomycin(#15140-122; Thermo Fisher Scientific)) with 1% RevitaCell™ Supplement (#A2644501; Thermo Fisher Scientific) for 24 h. Cells were kept in N2 medium for the next 10 days (d) of differentiation (d10) and 100 ng/mL of human Noggin(#130-103-456; Miltenyi Biotec), 10 µM of SB431542 (#130-106-543; Miltenyi Biotec) BMP/TGFβ inhibitors and 375 ng/mL of active human SHH (C24II) (#130-095-727; Miltenyi Biotec) were added from d0 to d9 (SB431542) or d10 (Noggin and SHH C24II) to this medium. Cells were treated with 100 ng/mL of human FGF8b (#130-095-740; Miltenyi Biotec) from d2, and 0.6 µM CHIR99021 (#130-106-539; Miltenyi Biotec) from d3 to d16 of this differentiation protocol. Cells were split using 0.5 mM EDTA/DPBS (w/o Ca, Mg) between d9 to d11 and cryopreserved at −150 °C in freezing medium (1:1 mixture of DMEM:F12 and Neurobasal, 1% N-2 supplement, 4% B-27 supplement without vitamin A (#12587001; Thermo Fisher Scientific) and 1% GlutaMAX, 10% DMSO) on d16 of their differentiation [13]. After passaging or thawing, pre-patterned ventral midbrain (VM)/mDA NSCs/NPCs were plated at a density of 2.6 × 10^3^ cells/cm^2^ in B27 medium (Neurobasal, 2% B-27 supplement without vitamin A, 1% GlutaMAX and 0.2% penicillin/streptomycin) with 1% RevitaCell™ Supplement (for 24 h) and supplemented with 20 ng/mL of human BDNF (#130-096-286; Miltenyi Biotec) and 0.2 mM L-ascorbic acid (#A4403; Sigma-Aldrich, Taufkirchen, Germany) from d10, and with 10 ng/mL of human GDNF (#212-GD-010; R&D Systems, Minneapolis, MN, USA), 500 µM dibutyryl-cyclic AMP (db-cAMP) (#D0627; Sigma-Aldrich) and 1 µM N-[(3,5-difluorophenyl)acetyl]-l-alanyl-2-phenyl]glycine-1, 1-dimethylethyl ester (DAPT) (#2634; Bio-Techne, Wiesbaden-Nordenstadt, Germany) from d16 for the remaining culture time. Composition of each medium is summarized in Appendix B. At d31 (QC No. 2) and d61 (QC No. 3) of this modified differentiation protocol, the pre-patterned VM/mDA NSCs/NPCs and differentiating mDA precursors and neurons were routinely tested by RT-qPCR for the expression of stage-, region- or neuronal cell-type-specific marker genes (Table 2).

### 2.7. Reverse Transcription and Quantitative Polymerase Chain Reaction (RT-qPCR)

Cells at the corresponding stages in 6-well plates were harvested by treatment with 0.5 mM EDTA/DPBS (w/o Ca, Mg; only hiPSCs) or Accutase for ~5 min and pelleting at 350× *g* for 4 min. Total RNA was isolated with the Monarch Total RNA Miniprep Kit (#T2010S; New England Biolabs) and 1 µg of total RNA was reverse transcribed using LunaScript^®^ RT SuperMix Kit (# M3010L; New England Biolabs), following the manufacturers’ instructions. PCR reactions were set up in duplicates in 96-well plates or 0.2 mL tubes using 1 µL of cDNA, 1 µM of the primer pairs listed in Table 2 (biomers.net GmbH, Ulm, Germany) and PowerUp™ SYBR™ Green Master Mix (#A25742; Thermo Fisher Scientific). Amplifications were performed on a StepOnePlus™ Real-Time PCR System (Thermo Fisher Scientific) with the following parameters: 30 s at 95 °C, 1 min at 60 °C for 40 cycles. Normalized ratios for each gene of interest were calculated by the formula 2^−dCt^, and normalized fold changes (relative to undifferentiated hiPSCs) were calculated by the 2^−ddCt^ method, in which dCt is the Ct value of the gene of interest minus the Ct value of the housekeeping gene *ACTB* (internal control) [27]. Data are from three technical replicates (independent differentiation experiments) [28].

### 2.8. Immunocytochemistry (ICC)

Cells were grown on 4-, 8- or 18-well µ-slides (ibidi, Gräfelfing, Germany) to the corresponding stages, rinsed twice in PBS pH 7.4 and fixed in 4% paraformaldehyde in PBS for 15 min, washed 3x in PBS for 5 min, permeabilized in 0.5% Triton X-100/PBS for 15 min and washed 3x in PBS for 5 min, all at room temperature. Cells were pre-incubated in 5% normal donkey serum (NDS) in permeabilization solution for 1 h at room temperature and incubated with primary antibodies (Table 3) overnight at 4 °C. After 3 × 10 min washes in PBS, cells were incubated with Alexa Fluor 488 and 555 secondary antibodies (Table 4) in 2.5% NDS in permeabilization solution for 1–2 h, washed 3 × 10 min with TBS (50 mM Tris-HCl pH 7.5; 0.9% NaCl) and incubated with 0.5 μg/mL of 4′,6-diamidino-2-phenylindole (DAPI) in PBS for 15 min, all at room temperature. After 3 × 5 min washes in TBS, cells were mounted in mounting medium (ibidi) and imaged on a DMi8 widefield microscope (Leica Microsystems, Wetzlar, Germany). Images were acquired with a DFC9000 GT sCMOS camera using LAS X Premium software (Leica Microsystems) and processed with Adobe Photoshop (Adobe, San Jose, CA, USA) software.

### 2.9. Cell Counting

Immunostained cells grown on 4-, 8- or 18-well µ-slides (ibidi) were visualized with a DMi8 (Leica Microsystems) widefield microscope equipped with brightfield, phase-contrast and fluorescence optics. Images were acquired with a DFC9000 GT sCMOS camera using LAS X Premium software (Leica Microsystems). For each well, three random fields were selected and photographed. Single-positive cells immunostained for TH, TUBB3, NESTIN and RFP (mScarlet), and counterstained with DAPI, were manually counted in each of these fields. Subsequently, the numbers of double-positive cells for the corresponding marker proteins were determined by the overlay images for each field. Cell numbers were averaged, and the percentage of TH-single-positive or TH- and TUBB3-double-positive cells among all DAPI^+^ or TUBB3^+^ cells, respectively, or the percentage of NESTIN and/or RFP single- and double-positive cells among all marker-positive cells was calculated and subjected to statistical analyses.

### 2.10. Enzyme-Linked Immunosorbent Assay (ELISA) for Catecholamines

Cells grown in 6-well plates at d51 of the modified differentiation protocol were treated as described by Kim et al. [29]. Briefly, cells were incubated in fresh B27 medium alone for 30 min and sequentially exposed to DPBS (w/o Ca, Mg) (basal condition) and modified Tyrode’s solution (119 mM NaCl, 2 mM CaCl_2_, 1.2 mM MgCl_2_-6 H_2_O, 10 mM glucose, 3.3 mM HEPES and 2.7 mM HEPES-Na^+^ salt; pH 7.4) with low (3 mM) and high (40 mM) potassium chloride (KCl) for 10 min each at 37 °C. Supernatants were collected and immediately mixed with 1 mM ascorbic acid and 1 mM EDTA to prevent DA auto-oxidation. Cells were detached with Accutase and lysed in lysis buffer (1% Triton X-100, 150 mM NaCl, 50 mM Tris, pH 8.0 and Halt™ Protease Inhibitor Cocktail (#87786; ThermoFisher Scientific)) to determine their total protein content by Bradford assay (#K015.1; Roth, Karlsruhe, Germany). Catecholamine (DA, noradrenaline and adrenaline) contents in the supernatants were determined using a TriCat ELISA (#RE59395; IBL International/Tecan, Hamburg, Germany) and an Emax Plus microplate reader (Molecular Devices, San Jose, CA, USA), according to the manufacturers’ protocols. The catecholamine contents were normalized to the total protein content in each sample.

### 2.11. Western Blot (WB)

Cells grown in 6-well plates from d7 to d61 of the modified differentiation protocol were lysed in lysis buffer (see Section 2.10) and boiled in 2× Laemmli buffer (62.5 mM Tris-HCl pH 6.8, 2% SDS, 25% glycerin, 0.01% bromophenol blue and 5% β-mercaptoethanol) at 95 °C for 5 min. Total proteins (1 µg per sample, determined by Bradford assay) were separated by 10% SDS-PAGE, blotted on Amersham™ Protran^®^ nitrocellulose membranes (#GE10600001; Sigma-Aldrich) and stripped, as described by [30]. Blots were probed with the primary antibodies and horseradish peroxidase (HRPO)-conjugated secondary antibodies listed in Table 3 and Table 4, respectively, developed in Pierce™ ECL Substrate (#32209; ThermoFisher Scientific) and imaged with a ChemiDoc Touch Imaging System (Bio-Rad Laboratories, Hercules, CA, USA).

### 2.12. Live Cell and Ca^2+^ Imaging

Cells grown in 6-well plates were directly visualized with a DMi8 widefield microscope (Leica Microsystems). Phase-contrast and fluorescent images were acquired with a DFC9000 GT sCMOS camera using LAS X Premium software (Leica Microsystems). For Ca^2+^ imaging, cells were grown in 35 mm µ-dishes (ibidi) until the desired stage. Before imaging, cells were rinsed once with recording medium (128 mM NaCl, 1 mM CaCl_2_, 1 mM MgCl_2_, 45 mM sucrose, 10 mM glucose and 10 mM HEPES; pH 7.4) and incubated in this medium with 4 µg/mL of Fluo-8-AM for 30 min at 37 °C, as described by Carola et al. [31]. Cells were washed once with recording medium to remove residual Fluo-8, replenished with fresh recording medium and immediately imaged on a DMi8 widefield microscope equipped with an on-stage incubator (Pecon/Leica Microsystems).

### 2.13. Flow Cytometry and Fluorescence-Activated Cell Sorting

Pre-patterned *wt* (XM001) and *NES*-mScarlet VM/mDA NSCs/NPCs grown in 6-well dishes at d11 and d23 of the modified differentiation protocol were harvested and dissociated in Accutase to obtain a single-cell suspension. Cells were resuspended in 2% human serum albumin (#9988; Irvine Scientific, Santa Ana, CA, USA) in DPBS (w/o Ca, Mg). Flow cytometry and fluorescence-activated cell sorting of mScarlet^+^ cells were performed with a BD FACSMelody™ Cell Sorter (BD Biosciences, Franklin Lakes, NJ, USA). The population of interest was identified through gating for singlets and cell size and granularity, based on the forward and side scatter of the *wt* control. A total of 30,000 events were acquired for each sample. Data were plotted with FlowJo™ software (LLC/BD Biosciences).

### 2.14. Statistical Analysis

Data are represented as the mean ± SEM from three or two independent experiments (technical replicates, [28]) for RT-qPCR and cell counting or ELISA, respectively. For statistical calculations, graphs were plotted and a two-sample *t*-test was conducted with a significance threshold of 95% (* *p* < 0.05) or 99.5% (** *p* < 0.005) using MATLAB software version R2021b. Non-significant results were not labelled in the graphs.

## 3. Results

### 3.1. Generation of NES-mScarlet Reporter hiPSCs

For the targeting of the human *NESTIN* (*NES*) gene, we constructed a recombination vector comprising a 2A self-cleaving peptide [22] and the *mScarlet* coding region [23] flanked by 1.5 kb homology regions located up- and downstream of the *NES* stop codon in exon 4 (Figure 1). Upon induction of a double-strand break in the *NES* locus of hiPSCs using Cas9 and a sgRNA recognizing the end of exon 4, the *P2A-mScarlet* coding region was inserted by homologous recombination upstream of the *NES* stop codon. The targeting vector was transfected into the XM001 hiPSCs, together with an expression vector for Cas9, sgRNA and RFP. Three days later, the transfected cells were enriched by fluorescent cell sorting, replated, subjected to a second cycle of transfection/sorting to increase the overall targeting efficiency and plated at low density for the isolation of single-cell-derived colonies. Isolated hiPSC colonies were further expanded and genomic DNA was analyzed by PCR for the detection of wildtype or targeted *NES* alleles. We characterized 24 colonies initially with primer pair P1/P2 able to detect the wildtype *NES* allele by a 474 bp band and genomic vector copies by a 1254 bp band (Figure 1).

**Figure 2:**
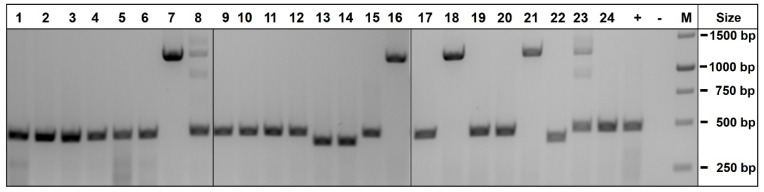
PCR genotyping of *NES*-mScarlet hiPSC clones using primer pair P1/P2. Genomic DNA from 24 XM001 hiPSC clones derived from transfection with Cas9/sgRNA and a *NES*-targeting vector was subjected to PCR amplification using primers P1/P2. The wildtype *NES* locus yielded a band of 474 bp, whereas a 1254 bp band was amplified from the targeting vector in clones #7, #8, #16, #18, #21 and #23. +, untransfected control DNA; −, water control; M, size marker.

Six of the tested clones exhibited the vector-specific band, either together with the wildtype band (clones #8 and #23) or did not exhibit the wildtype band (clones #7, #16, #18 and #21), suggesting that both *NES* alleles were targeted in the latter group (Figure 2). For the further genotyping of these clones, we designed primers P5/P6 and P7/P8 for amplification of the targeted loci using an *mScarlet* primer binding site located within the vector and a genomic primer outside of the vector homology regions. This analysis revealed that all of the 6 clones exhibited the 1659 bp band predicted from the upstream region of the targeted *NES* locus (P5/P6, Figure 1) and 5 clones (except for clone #8) showed the predicted 1855 bp band covering its downstream region (P7/P8, Figure 1) (Figure S1). PCR products were Sanger-sequenced and compared to the sequence of the targeted *NES* locus upon recombination with the *NES-mScarlet* targeting vector. This analysis confirmed the presence of correctly targeted alleles in clones #7, #18, #21 and #23. Due to the absence of the *NES* wildtype band, clones #7, #18 and #21 were homozygously targeted, whereas clone # 23 harbored one targeted and one wildtype allele. Karyotyping using an Illumina platform for SNP analysis confirmed normal karyotypes for clones #18, #21 and #23, whereas clone #7 showed a duplication on chromosome 1 and was excluded from further analysis (Figure S2). In the remaining three clones (#18, #21 and #23), the set of six predicted off-target sites showing one or two mismatches to the sgRNA target sequence were analyzed and confirmed to be unaltered (Figure S3).

### 3.2. NES-mScarlet hiPSCs Retain Expression of Pluripotency Marker Genes

**Figure 3:**
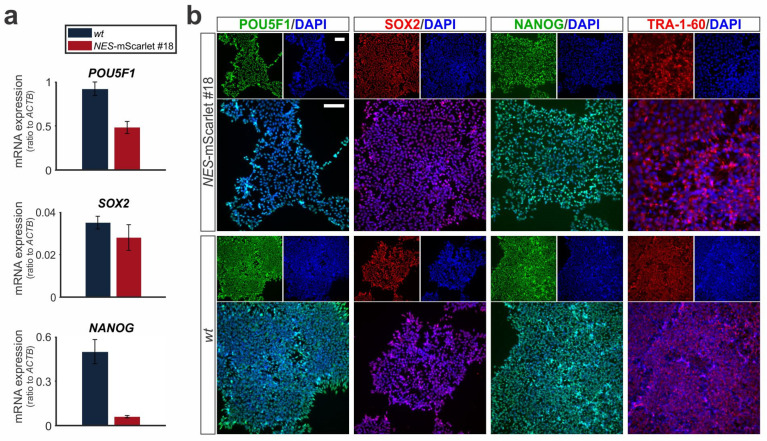
CRISPR-Cas9-targeted *NES*-mScarlet hiPSCs retain their pluripotency. (**a**) RT-qPCR for the pluripotency marker genes *POU5F1* (*OCT4*), *SOX2* and *NANOG* on total RNA isolated from *wt* (XM001) or *NES*-mScarlet #18 hiPSCs. The values shown are the 2^−dCt^ ratios of the difference between cycle thresholds (dCt) of the corresponding marker genes and the housekeeping gene *ACTB*; *n* = 3 independent differentiation experiments; (**b**) ICC for the detection of the pluripotency marker proteins POU5F1 (OCT4), SOX2, NANOG and TRA-1-60 in the *NES*-mScarlet #18 and *wt* hiPSCs. Top panels are the single green (POU4F1 and NANOG) or red (SOX2 and TRA-1-60) and blue (DAPI) channel views of the merged images below. Scale bars: 100 µm.

After the initial expansion of the three correctly targeted *NES*-mScarlet hiPSC clones #18, #21 and #23, we further characterized these cells based on their morphology, cell growth, pluripotency and reporter expression. Because we did not detect the red mScarlet reporter by direct visual inspection under a fluorescence microscope in the heterozygote clone #23, we discarded this hiPSC clone from further analyses (see also Section 3.5). The better growth properties of *NES*-mScarlet hiPSC clone #18 (henceforth named *NES*-mScarlet #18) compared to clone #21 (both homozygous for the targeted locus; data not shown) let us characterize clone #18 in more depth. To determine whether the CRISPR-Cas9-targeted and expanded (passage 31) *NES*-mScarlet hiPSCs retained their pluripotent properties, the expression of the pluripotency genes *POU5F1* (*OCT4*), *SOX2* and *NANOG* was determined in these cells by RT-qPCR (QC No. 1) and ICC. Transcription of *POU5F1* (*OCT4*), *SOX2* and *NANOG* in the *NES*-mScarlet #18 hiPSCs was comparable to the parental XM001 *wt* hiPSCs, and no statistically significant difference was detected between these two cell lines (Figure 3a). Moreover, the corresponding proteins POU5F1 (OCT4), NANOG and SOX2, as well as the additional human-specific and cell surface marker TRA-1-60, were expressed in all targeted cells to a similar extent as in the *wt* hiPSCs (Figure 3b). We thus concluded that the newly generated *NES*-mScarlet hiPSCs (clone #18) retained their normal growth characteristics and pluripotent properties.

### 3.3. NES-mScarlet hiPSCs Differentiate into Ventral Midbrain Human Neural Stem and Progenitor Cells

**Figure 4:**
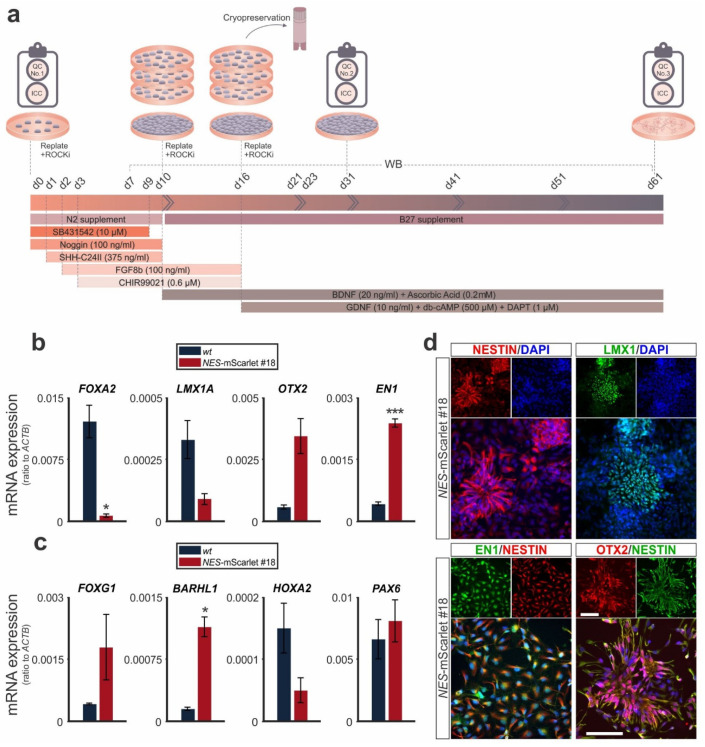
*NES*-mScarlet hiPSCs differentiate into genuine VM NSCs/NPCs. (**a**) Schematic depiction of the modified differentiation protocol, including the timeline of culture media addition and factor incubation, as well as the RT-qPCR, ICC and WB analyses performed in this context; (**b**) RT-qPCR for the VM marker genes *FOXA2*, *LMX1A*, *OTX2* and *EN1* on total RNA isolated from *wt* (XM001) or *NES*-mScarlet #18 hiPSC-derived cells on d31 of the modified differentiation protocol; (**c**) RT-qPCR for the forebrain (*FOXG1*), ventral diencephalic (*BARHL1*), hindbrain (*HOXA2*) and diencephalic/neural rosette (*PAX6*) marker genes on total RNA isolated from *wt* (XM001) or *NES*-mScarlet #18 hiPSC-derived cells on d31 of the modified differentiation protocol. The values shown in (**b**) and (**c**) are the 2^−dCt^ ratios of the difference between cycle thresholds (dCt) of the corresponding marker genes and the housekeeping gene *ACTB*; * *p* < 0.05, *** *p* < 0.001 in the two-sample *t*-test, *n* = 3 independent differentiation experiments; (**d**) ICC for the detection of the VM NSC/NPC marker proteins NESTIN, LMX1, EN1/NESTIN and OTX2/NESTIN in the *NES*-mScarlet #18 hiPSC-derived cells on d31 of the modified differentiation protocol. Top panels are the single red (NESTIN and OTX2), green (LMX1, EN1 and NESTIN) and blue (DAPI) channel views of the merged images below. Scale bars: 100 µm.

To determine their ability to generate human NSCs/NPCs and their potential as reporter cells for this cell type and stage, we differentiated the targeted *NES*-mScarlet hiPSCs under defined conditions into NSCs/NPCs of a specific brain region, the VM. To this end, we modified the previously published monolayer protocols [13,25,26] by monitoring the expression of specific marker genes for VM NSCs/NPCs (Table 2) in the differentiating cells (Figure 4a). The simultaneous differentiation of the parental *wt* (XM001) hiPSC line under the same conditions was used as a control. The identity of the generated NSCs/NPCs was determined through the detection of these genes and proteins by RT-qPCR and ICC on d31 of the modified differentiation protocol, after passaging and, eventually, cryopreservation of the cells (QC No. 2; Figure 4a). The transcription of the VM marker genes *FOXA2*, *LMX1A*, *OTX2* and *EN1* was robustly induced in the *NES*-mScarlet #18-derived cells, similar to their *wt* counterparts, at d31 of the modified protocol (Figure 4b). The mRNA levels of the ventral marker *FOXA2* were significantly lower (*p* = 0.0456), and the transcription of the midbrain marker *EN1* was significantly higher (*p* = 0.0008), in the *NES*-mScarlet #18-derived cells compared to their *wt* counterparts, whereas *OTX2* and the VM- and mDA-specific marker gene *LMX1A* did not show any significant differences between both hiPSC lines.

To exclude that the generated NSCs/NPCs had acquired an alternative fate of a different brain region (fore- or hindbrain), we also monitored the expression of a forebrain-specific (*FOXG1*), two diencephalic (*BARHL1* and *PAX6*) and a hindbrain-specific (*HOXA2*) marker gene in the differentiating cells at d31. Except for the diencephalic and neural rosette marker *PAX6*, which was expressed at relatively high levels in both the *NES*-mScarlet #18 and *wt* NSCs/NPCs, these cells exhibited much lower mRNA ratios of the forebrain marker *FOXG1*, the diencephalic marker *BARHL1* and the hindbrain marker *HOXA2* compared to the midbrain markers *EN1* and *OTX2* (Figure 4c). The transcription of the ventral diencephalic marker *BARHL1* was significantly higher (*p* = 0.016) in the *NES*-mScarlet #18 cells compared to the *wt* cells.

To confirm their identity as VM NSCs/NPCs, we assessed the expression of the NSC marker proteins NESTIN and SOX2, alone or together with the midbrain markers EN1, OTX2 and the VM and mDA-specific marker LMX1, by ICC on the differentiating cells (Figure 4d and Figure S4). NESTIN and SOX2 were expressed in almost all *NES*-mScarlet #18-derived cells, and NESTIN co-localized with EN1 or OTX2 in most cell nuclei at d31 of the modified differentiation protocol (Figure 4d and Figure S4). The expression of the VM and mDA-specific marker LMX1, by contrast, was restricted to a subset of the differentiating cells at this stage of the protocol, consistent with the lower *LMX1A* mRNA ratios in these cultures (Figure 4b,d and Figure S4). We concluded that despite some variabilities in the mRNA levels of certain brain-region- and cell-type-specific marker genes between the *NES*-mScarlet #18-derived NSCs/NPCs and their *wt* counterparts, the generated *NES*-mScarlet hiPSCs (clone #18) are capable of differentiating under defined culture conditions into *bona fide* NSCs/NPCs with a VM identity.

### 3.4. NES-mScarlet hiPSCs Differentiate into Mature Midbrain Dopaminergic Neurons

**Figure 5:**
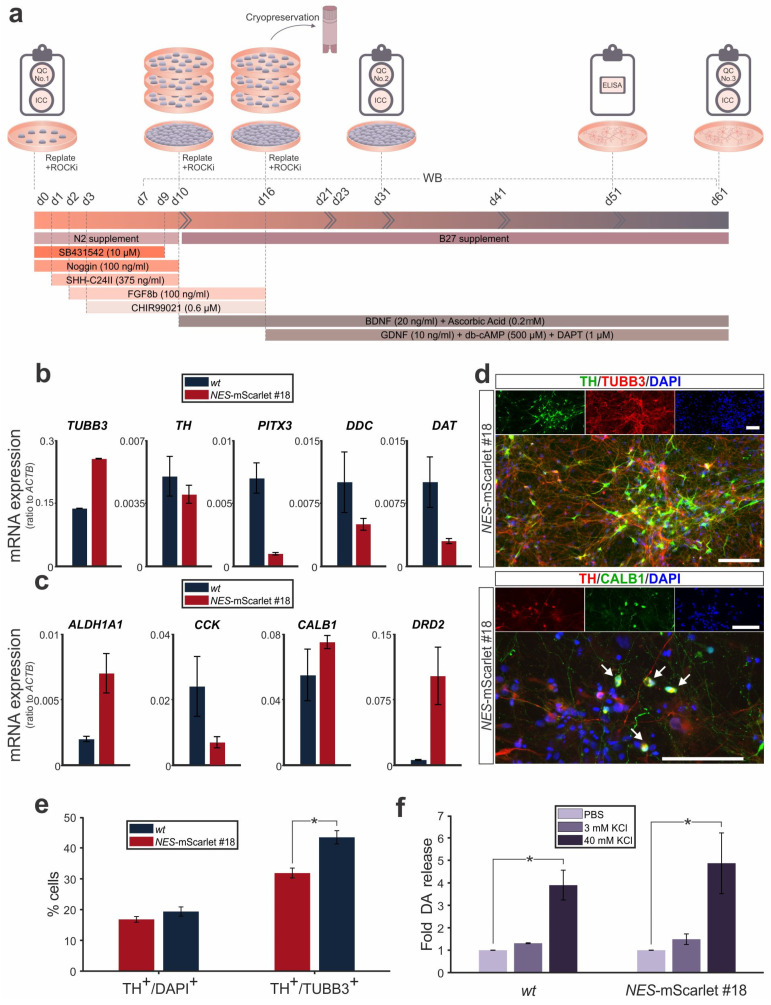
*NES*-mScarlet hiPSCs differentiate into mature mDA neurons. (**a**) Schematic depiction of the modified differentiation protocol, including the timeline of culture media addition and factor incubation, as well as the RT-qPCR, ICC, WB and ELISA analyses performed in this context; (**b**) RT-qPCR for the generic neuron marker gene *TUBB3* and the generic mDA marker genes *TH*, *PITX3*, *DDC* and *DAT* (*SLC6A3*) on total RNA isolated from *wt* (XM001) or *NES*-mScarlet #18 hiPSC-derived cells on d61 of the modified differentiation protocol; (**c**) RT-qPCR for the subset-specific mDA marker genes *ALDH1A1* and *DRD2* (SNc DA neurons) or *CCK* and *CALB1* (VTA DA neurons) on total RNA isolated from *wt* (XM001) or *NES*-mScarlet #18 hiPSC-derived cells on d61 of the modified differentiation protocol. The values shown in (**b**) and (**c**) are the 2^−dCt^ ratios of the differences between the cycle thresholds (dCt) of the corresponding marker genes and the housekeeping gene *ACTB*; *n* = 3 independent differentiation experiments; (**d**) ICC for the detection of the generic neuron marker TUBB3 and the mDA marker TH, as well as the VTA DA-specific marker CALB1 in the *NES*-mScarlet #18 hiPSC-derived cells on d61 of the modified differentiation protocol. Top panels are the single green (TH and CALB1), red (TUBB3 and TH) and blue (DAPI) channel views of the merged images below. Arrows point at TH^+^/CALB1^+^ double-positive cells. Scale bars: 100 µm; (**e**) Quantification of TH^+^/DAPI^+^ and TH^+^/TUBB3^+^ double-positive cells among all DAPI^+^ or TUBB3^+^ cells in the *wt* (XM001) or *NES*-mScarlet #18 hiPSC-derived cultures on d61 of the modified differentiation protocol; * *p* < 0.05 in the two-sample *t*-test, *n* = 3 independent differentiation experiments; (**f**) Fold change of DA release (relative to the PBS-treated cells, set as 1) after treatment of the *wt* (XM001) or *NES*-mScarlet #18 hiPSC-derived cells with 3 or 40 mM KCl on d51 of the modified differentiation protocol; * *p* < 0.05 in the one-sample *t*-test, *n* = 2 independent differentiation experiments.

Subsequently, we tested whether the targeted *NES*-mScarlet hiPSCs were also capable of generating mature mDA neurons using the modified differentiation protocol (Figure 5a). Again, we used the simultaneous differentiation of the parental *wt* (XM001) hiPSC line under the same conditions as a control. The identity of the generated mDA neurons was determined through detection of both generic and subset-specific marker genes and proteins for this particular neuronal cell type by RT-qPCR and ICC on d61 of the modified differentiation protocol, which was the last time point analyzed in this context (QC No. 3; Figure 5a).

The transcription of the generic neuronal marker gene *TUBB3* was strongly induced in the *NES*-mScarlet #18 hiPSC-derived cultures, similar to their *wt* counterparts, at d61 of the modified protocol (Figure 5b). The generic mDA marker genes *TH*, *PITX3*, *DDC* and *DAT* (*SLC6A3*), by contrast, were expressed at lower ratios in the mature cells, suggesting that only a smaller proportion of all neurons in the culture dish had differentiated into the mDA cell type (Figure 5b). No significant differences in the expression levels (ratios) of these generic mDA marker genes were detected between the *NES*-mScarlet #18 hiPSC-derived cells relative to their *wt* counterparts. Although similar variabilities in the mRNA ratios of the subset-specific mDA marker genes *ALDH1A1*, *DRD2*, *CCK* and *CALB1* were observed between the *wt* (XM001) and *NES*-mScarlet #18 hiPSC-derived cells at this stage, none of them reached statistical significance (Figure 5c). Of note, the SNc DA marker gene *DRD2* and the VTA DA marker gene *CALB1* were transcribed at higher ratios, whereas the SNc DA-specific marker *ALDH1A1* and the VTA DA-specific marker *CCK* were expressed at lower ratios in the *NES*-mScarlet #18 hiPSC-derived cells at d61 of the modified protocol (Figure 5c). It, thus, remains to be established whether this reflects a true mDA neuron subtype-specific differentiation effect or whether these variances in gene expression levels are due to technical issues, such as distinct primer binding and amplification efficiencies.

Next, we assessed the expression of some of these marker proteins in the mature cells. The DA neuron marker TH was detected in a subset of the TUBB3-positive neurons derived from the *NES*-mScarlet #18 hiPSCs on d61 of their differentiation, thus confirming the previous RT-qPCR data (Figure 5d). Only very few of the TH-positive *NES*-mScarlet #18-derived DA neurons co-expressed the VTA DA marker CALB1 at this stage (Figure 5d). Quantification of the proportion of TH-positive cells among all DAPI-positive cells revealed that around a fifth (16.82% ± 0.95 for *wt* (XM001) and 19.37% ± 1.5 for *NES*-mScarlet #18) had acquired the DA neuron identity, whereas the proportion of TH-positive DA cells reached around one third to almost a half (31.86% ± 1.57 for *wt* (XM001) and 43.43% ± 2.15 for *NES*-mScarlet #18) of the TUBB3-positive neurons on d61 of the modified protocol (Figure 5e). Notably, the proportion of TH- and TUBB3-double-positive DA neurons was significantly higher in the *NES*-mScarlet #18-derived cultures compared to the *wt* (XM001) cultures (Figure 5e).

Additionally, we determined the ability of the mature mDA neurons (d51 of the modified protocol) to release DA under both unstimulated conditions (PBS treatment) and after stimulation with low (3 mM) or high (40 mM) KCl concentrations [32]. Upon low KCl treatment, DA release from the differentiated cells increased by almost 1.5-fold (1.31 ± 0.02-fold for *wt* (XM001) and 1.49 ± 0.23-fold for *NES*-mScarlet #18), whereas high KCl treatment led to an approx. 4 to 5-fold (3.90 ± 0.66-fold for *wt* (XM001) and 4.87 ± 1.35-fold for *NES*-mScarlet #18) and significant release of DA relative to the unstimulated condition (Figure 5f). Other catecholamines downstream of DA synthesis (noradrenaline and adrenaline) were not released at detectable levels from the unstimulated and stimulated cells (data not shown). These data indicated that the targeted *NES*-mScarlet hiPSCs, similar to the parental *wt* (XM001) hiPSCs, were capable of generating mature (based on their marker gene expression profile) and functional (based on their ability to release DA under both basal conditions and after KCl stimulation) mDA neurons using a modified monolayer protocol for the directed differentiation of these cells.

### 3.5. NES-mScarlet hiPSCs Are Accurate Reporter Cells of Human Neural Stem/Progenitor Cells

**Figure 6:**
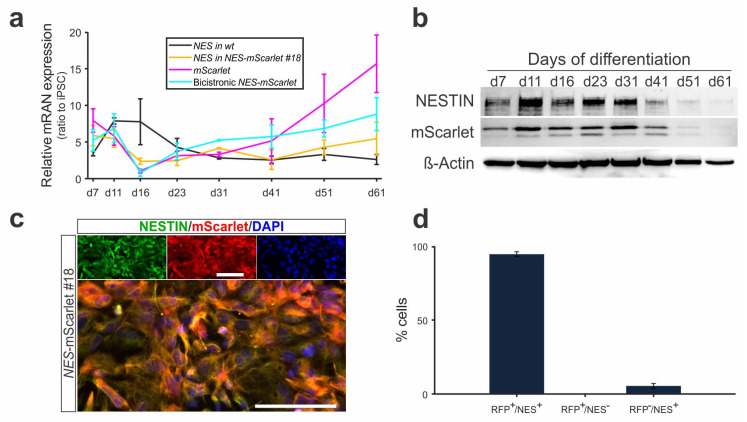
*NES*-mScarlet hiPSCs are accurate reporters of human NSCs/NPCs. (**a**) Time course of *NES*, *mScarlet* and bicistronic *NES-mScarlet* mRNA detection in differentiating *wt* (XM001) and *NES*-mScarlet #18 hiPSCs, determined by RT-qPCR on d7, d11, d16, d23, d31, d41, d51 and d61 of the modified differentiation protocol. The values shown are the fold changes (2^−ddCt^ ratios) of the differences between the cycle thresholds (dCt) of the corresponding amplicon and the housekeeping gene *ACTB* relative to undifferentiated hiPSCs, *n* = 3 independent differentiation experiments; (**b**) Western blot for the detection of full-length human NESTIN (~240 kDa) and mScarlet (~30 kDa) proteins in cell lysates from differentiating *NES*-mScarlet #18 hiPSCs on d7, d11, d16, d23, d31, d41, d51 and d61 of the modified differentiation protocol. Beta-actin (42 kDa) was used as a loading control; (**c**) ICC for the detection of the NSC/NPC marker protein NESTIN and the mScarlet reporter in the *NES*-mScarlet #18 hiPSC-derived cells on d23 of the modified differentiation protocol. Top panels are the single green (NESTIN), red (mScarlet) and blue (DAPI) channel views of the merged image below. Scale bars: 100 µm; (**d**) Quantification of mScarlet (RFP) and/or NESTIN (NES) single- and double-positive cells in the *NES*-mScarlet #18 hiPSC-derived cultures on d23 of the modified differentiation protocol, *n* = 3 independent differentiation experiments.

Given the previous results, we determined the time course and efficacy of NESTIN and mScarlet expression from the CRISPR-Cas9-targeted *NES* locus in the differentiating *NES*-mScarlet #18 hiPSCs and compared it to the parental *wt* (XM001) hiPSC line in the case of NESTIN. The targeted *NES* locus was expected to produce a bicistronic mRNA encoding the *NES* and *mScarlet* open reading frames, which gives rise to two independent proteins (NESTIN and mScarlet) upon 2A-mediated “self-cleavage” (ribosome skipping) during translation. RT-qPCR to detect *NES*, *mScarlet* and bicistronic *NES-mScarlet* mRNA sequences revealed that the mRNA ratios increased notoriously and peaked at d7–d11 of our modified differentiation protocol relative to the undifferentiated *NES*-mScarlet #18 and *wt* hiPSCs (Figure 6a), indicating that these cells had acquired an NSC/NPC (neuroectodermal) identity during the first week of differentiation. After this time point (d11), the levels of *NES*, *mScarlet* and bicistronic *NES-mScarlet* mRNA in the differentiating *NES*-mScarlet #18 NSCs/NPCs decreased strongly to almost the same levels in the undifferentiated hiPSCs at d16 (Figure 6a), which coincided with the end of patterning factor treatment and the initiation of terminal differentiation in the modified protocol (Figure 5a). Thereafter, *NES* mRNA ratios stayed at these low levels over the remaining culture time and increased only slightly towards the end (around d61) of the differentiation protocol in the *NES*-mScarlet #18 but not in the *wt* hiPSCs (Figure 6a). By contrast, the ratios of *mScarlet* and bicistronic *NES-mScarlet* mRNA increased by d23 and remained stable at these higher levels over the next ~20 days but appeared to increase slightly again towards the end (d51–d61) of the modified differentiation procedure (Figure 6a). We concluded that the targeted *NES* locus generated a bicistronic *NES-mScarlet* mRNA whose expression accurately recapitulated the acquisition of an NSC/NPC identity by the differentiating *NES*-mScarlet #18 hiPSCs and their further maturation into postmitotic mDA neurons. The slight increase of bicistronic *NES-mScarlet* mRNA levels towards the end of the differentiation procedure might indicate the persistence of a small but detectable number of self-renewing NSCs/NPCs in the mature cultures (see below). The measured differences in ratios (2^−ddCt^ values) between the amplified *NES*, *mScarlet* and *NES-mScarlet* sequences are most likely due to different amplification efficiencies for the three primer pairs used in this experiment.

We next determined the absence of a potential fusion protein (due to missed ribosome skipping during translation) and the pattern of NESTIN and mScarlet protein expression in the differentiating *NES*-mScarlet #18 hiPSCs. Throughout the protocol, we only detected a high molecular weight band around 240 kDa, which is the expected size of the full-length human NESTIN protein [14], and a smaller band around 30 kDa, which corresponds to the expected size of the monomeric Scarlet protein (26.4 kDa, [23]), in the differentiating cells (Figure 6b and Figure S5). Because we did not detect any higher molecular weight band that might correspond to a fusion of these two proteins, we concluded that the NESTIN intermediate filament and mScarlet reporter were correctly processed into single proteins in the targeted *NES*-mScarlet #18 hiPSCs. Notably, the levels of NESTIN and mScarlet proteins in these cells also appeared to increase between d7 and d11 of the modified differentiation protocol, corresponding to the NSC/NPC stage (Figure 6b). After this time point (d11), they appeared to remain at the same levels between d16 and d31 (postmitotic precursor cell stage) but dropped noticeably at d41 and became almost undetectable at d61 (mature neuron stage) of this protocol (Figure 6b). This finding is in line with the RT-qPCR data (Figure 6a) and indicated that both NESTIN and mScarlet protein levels are downregulated to the same extent and at the same time that the *NES*-mScarlet #18 NSCs/NPCs acquire a mature (mDA) neuron identity.

We then assessed the fidelity of the differentiating *NES*-mScarlet #18 hiPSCs as a reporter for human NSCs/NPCs. Therefore, we double-labeled the differentiating cells by ICC for NESTIN and mScarlet proteins at d23 of the modified protocol (Figure 6c). This corresponds to a time point when bicistronic *NES-mScarlet* mRNA, as well as NESTIN and mScarlet protein levels, are already declining in the *NES*-mScarlet #18 NSCs/NPCs due to their differentiation into postmitotic (VM and mDA) precursors (Figure 6a,b), thus allowing for the distinction between NESTIN- and/or mScarlet-positive or -negative cells. In fact, 94.54% ± 1.63 of the NESTIN-positive cells also expressed mScarlet (i.e., they were double-positive cells), and only 5.46% ± 1.63 of the NESTIN-positive cells were mScarlet-negative, thus confirming that the vast majority of the NESTIN-positive *NES*-mScarlet #18 NSCs/NPCs co-expressed the reporter protein mScarlet on d23 of their differentiation (Figure 6d). Importantly, we did not detect any mScarlet-positive but NESTIN-negative cells in our cultures, indicating that the mScarlet reporter was not ectopically expressed in the *NES*-mScarlet #18 hiPSC-derived cells (Figure 6d). Together with the WB data, we could also rule out the persistence of mScarlet protein in NESTIN-negative cells due to different turnover kinetics for the two proteins.

Lastly, we evaluated the expression of the red fluorescent reporter in living *NES*-mScarlet #18 hiPSC-derived cells during the course of differentiation (Figure S6). Faint mScarlet-positive cells were detected as early as d3 of the modified protocol in the differentiating *NES*-mScarlet #18 and *NES*-mScarlet #21 cultures, both homozygous for the targeted locus, by direct visualization under a fluorescence microscope (Figure S6 and data not shown). The intensity of the red mScarlet fluorescence, as well as the numbers of reporter-positive cells, increased during the subsequent days of their differentiation into NSCs/NPCs but gradually diminished as these cells become mature neurons after d31 of the modified protocol (Figure S6 and data not shown). By contrast, we could not detect any red mScarlet fluorescence in the heterozygote *NES*-mScarlet #23 hiPSC-derived cultures upon visual inspection under a fluorescence microscope, suggesting that one *NES*-mScarlet allele in the targeted cells was not sufficient for the direct (live) visualization of the mScarlet reporter (data not shown). We concluded that the *NES*-mScarlet #18 hiPSC line is a sufficiently accurate reporter cell line for NESTIN expression in human NSCs/NPCs, which can be visualized directly under a fluorescence microscope.

### 3.6. NES-mScarlet hiPSCs Are Suitable for Live Cell Imaging and Flow Cytometric Analysis and Cell Sorting

**Figure 7:**
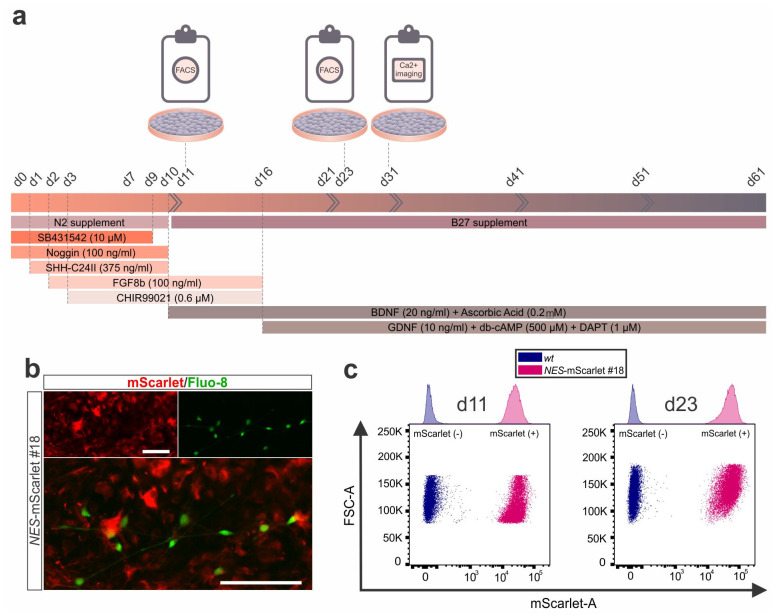
Live cell/Ca^2+^ imaging and flow cytometric analysis of *NES*-mScarlet hiPSC-derived NSCs/NPCs. (**a**) Schematic depiction of the modified differentiation protocol including the timeline of culture media addition and factor incubation as well as the Ca^2+^ imaging and fluorescence-based flow cytometric analyses and sorting (FACS) performed in this context; (**b**) Live cell imaging for the detection of red fluorescent mScarlet^+^ NSCs/NPCs and the green fluorescent Ca^2+^ indicator Fluo-8 in *NES*-mScarlet #18 hiPSC-derived cells on d31 of the modified differentiation protocol. Top panels are the single red (mScarlet) and green (Fluo-8) channel views of the merged image below. Scale bars: 100 µm; (**c**) Scatter plots of the flow cytometric analysis of *NES*-mScarlet #18 hiPSC-derived NSCs/NPCs (pink) and their *wt* (XM001) counterparts (control; blue) on d11 and d23 of the modified differentiation protocol. FSC-A, amplitude of the forward scatter signal; mScarlet-A, amplitude of the mScarlet red fluorescence. Top graphs are the corresponding histograms.

The original purpose of generating the *NES*-mScarlet #18 hiPSCs was the ability to combine these red fluorescent reporter cells for human NSCs/NPCs with other fluorescent (e.g., green) molecules or reporter proteins in live cell imaging, as well as the fluorescence-based flow cytometric analysis and sorting of these cells. To demonstrate the suitability of this reporter hiPSC line for combined fluorescence in live cell imaging, we used the green fluorescent Ca^2+^ indicator Fluo-8 to detect Ca^2+^-mediated activities in the *NES*-mScarlet #18 hiPSC-derived cells on d31 of the modified differentiation protocol (Figure 7a). Both the green fluorescence of the Fluo-8 Ca^2+^ indicator and the red fluorescence of the mScarlet reporter in NSCs/NPCs were easily distinguishable by live cell imaging of the differentiating cultures (Figure 7b). Nevertheless, the slow Ca^2+^ transients that were observed in the cultures at this stage of their differentiation were mostly restricted to the mScarlet-negative postmitotic neuronal precursor cells present in the dish (data not shown).

Next, fluorescence-based flow cytometry was performed with differentiating *wt* (XM001; control) and *NES*-mScarlet #18 cells on d11 and d23 of the modified protocol (Figure 7a). Differentiation d11 corresponded with the NSC/NPC stage and peak expression of both NESTIN and the mScarlet reporter, whereas d23 represented a later stage at which bicistronic *NES-mScarlet* mRNA levels were already declining and a few NESTIN-positive but mScarlet-negative cells were detected in the differentiating cultures (Figure 6). At both time points, more than 99% of the analyzed cells expressed the red fluorescent mScarlet reporter (99.95% at d11 and 99.96% at d23), indicating that the NSC/NPC reporter population can be sorted to a very high purity at the relevant stages using this approach (Figure 7c and Figure S7). Altogether, we concluded that the *NES*-mScarlet #18 hiPSCs are a reliable and accurate reporter cell line for human NSCs/NPCs that can be used in combination with other fluorophores for the direct identification or isolation of these cells in live cell imaging or fluorescence-based flow cytometric analyses and sorting assays. Moreover, the *NES*-mScarlet #18 NSCs/NPCs can be differentiated into mature and functional mDA neurons to the same extent as their parental (XM001) hiPSC line, as one example for a downstream application of these cells.

## 4. Discussion

We report the successful generation of a novel red fluorescent reporter hiPSC line (*NES*-mScarlet #18) for the direct detection in the culture dish and isolation to a high purity of human NSCs/NPCs. This was achieved by the introduction of a 2A self-cleaving peptide followed by the mScarlet reporter at the C-terminus of the well-characterized stem cell marker protein NESTIN [14], using CRISPR-Cas9-mediated homology-directed repair [33]. We chose this targeting strategy of the *NES* locus for several reasons. Firstly, two green fluorescent NESTIN reporter PSC lines are already available in the human context [17,18], but to our knowledge, a corresponding red fluorescent reporter PSC line has not been generated so far. The new *NES*-mScarlet hiPSC line, thus, expands the current tool set of human NESTIN reporter cell lines. The mScarlet fluorophore is an extremely bright (high fluorescence lifetime and quantum yield) and truly monomeric (no accumulation in organelles) RFP that was selected based on these particular spectral and biochemical as well as additional properties, including its tolerance to acidic environments and very low cytotoxicity [23]. Secondly, our approach avoided the synthesis of an unphysiological NESTIN-reporter fusion protein (as in the CRISPR-Cas9-targeted green fluorescent hiPSC line KSCBi005-A-1, [17]) and allowed the stoichiometric production of independent NESTIN and mScarlet proteins in the targeted cells. Thirdly, targeting the endogenous *NES* locus in the hiPSCs circumvented the problems associated with the generation of transgenic hiPSC lines using the well characterized second intron of the human *NES* gene [18,34]. These include restriction to the neural lineage and random integration of the transgene into the cell’s genome at variable copy numbers, often resulting in ectopic or silenced expression of the reporter due to positional effects [14,18]. Lastly, the absence of a selection marker in the targeted *NES* locus ensured the unperturbed transcription and translation from this locus. As expected, the *NES*-mScarlet hiPSCs retained a normal karyotype and maintained their pluripotency over several rounds of expansion.

We subsequently devised a modified protocol from published versions [13,25,26] to direct the newly generated *NES*-mScarlet hiPSCs into the VM NSC/NPC fate. Using this paradigm, the *NES*-mScarlet hiPSCs differentiated robustly and reproducibly into NSCs/NPCs of a VM identity, which was determined by their VM-specific marker gene and protein expression. Moreover, the pre-patterned VM NSCs/NPCs were capable of generating fully mature and functional mDA neurons, characterized by the expression of generic and subset-specific mDA marker genes and proteins, and by the release of DA under basal conditions and upon KCl stimulation. The numbers of TH-positive DA cells (~20%) or neurons (30–45%) in these cultures were within the expected range (7–70% around d50 of differentiation) and corresponded well with the published data [8,35]. However, the mRNA levels of the mDA-specific marker *LMX1A* were rather low compared to the other VM-specific genes, and this protein was only detected in a subset of the *NES*-mScarlet hiPSC-derived VM NSCs/NPCs. Furthermore, unwanted forebrain (*FOXG1*, *BARHL1*, *PAX6*) and hindbrain (*HOXA2*) marker genes were still expressed at detectable levels in the differentiating cells using this modified protocol. Both observations are in line with a newer report showing that a bi-phasic modulation of WNT signaling (via CHIR99021, initially low and later high) is necessary during the initial steps of human PSC conversion to a VM/mDA neural fate for the generation of *bona fide* VM/mDA NSCs/NPCs expressing high levels of *LMX1A* and low to absent levels of the aforementioned marker genes for other brain regions [29]. Conversely, the mature mDA neurons derived from the human VM/mDA NSCs/NPCs with this modified WNT signaling protocol exhibited rather low mRNA levels for both generic and subset-specific markers of these neurons [29], suggesting that the high WNT signaling levels in the second phase of this paradigm suppressed the generation of genuine and possibly subset-specific (SNc or VTA) mDA neurons. In fact, it was recently shown that inhibition of WNT signaling during the late phase of VM/mDA NSC/NPC patterning (after initially higher WNT signaling levels compared to our protocol [26]) promotes the preferential derivation of SNc DA neurons from human PSCs, whereas high WNT signaling levels during this phase force them into the VTA DA neuron identity [36]. We therefore speculate that an improved protocol, which precisely recapitulates the time course of WNT signaling during mDA neuron development in vivo [12,37] might be able to direct our *NES*-mScarlet hiPSCs into a genuine and possibly also subtype-specific mDA neuron identity. This will be assessed in future experiments.

We show that the new *NES*-mScarlet #18 hiPSC line is an accurate red fluorescent reporter cell line for human NSCs/NPCs and potentially also for all kinds of NESTIN-positive stem and progenitor cells. Using the modified protocol mentioned above, we demonstrate that the *NES*-mScarlet #18 hiPSCs reliably and reproducibly up- or downregulate the red fluorescent mScarlet reporter in the same temporal pattern as NESTIN is expressed from the native *NES* promoter during the course of their differentiation. Manual cell counting revealed that most (~95%) of the NESTIN-positive *NES*-mScarlet #18 hiPSC-derived NSCs/NPCs at d23 of the modified protocol co-expressed the mScarlet reporter. The few (~5%) NESTIN-positive but mScarlet-negative cells detected in this experimental paradigm might have been assigned erroneously to this category because of the weak to very weak expression of the reporter protein in these cells. Indeed, fluorescence-based flow cytometry of the *NES*-mScarlet #18 hiPSC-derived cultures on the same day (d23) of the modified differentiation protocol revealed that more than 99% of these cells are mScarlet-positive and can, thus, be sorted to a very high degree of purity. Most importantly, we were unable to detect any ectopic mScarlet-positive but NESTIN-negative cells in the manual cell counting paradigm, and Western blotting confirmed these data. We can, therefore, conclude that the expression of the red fluorescent mScarlet reporter faithfully recapitulates the expression of endogenous NESTIN in our new *NES*-mScarlet #18 hiPSC line, thus circumventing the problems associated with other NESTIN reporter lines. These include the ectopic expression of the reporter protein in transgenic PSC lines, probably because of positional effects after random integration of the transgene [18], and the prolonged detection of the green fluorescent reporter protein because of its low turnover in the cells [14,17].

Finally, we show that the *NES*-mScarlet #18 hiPSC line is a versatile tool for the direct identification and tracking of human NSCs/NPCs in the culture dish and their fluorescence-based flow cytometric analysis and sorting, which can be easily combined with other fluorophores of distinct spectral properties, e.g., in the blue, green or far-red range. Here, we used the red mScarlet fluorescence and the green Ca^2+^ indicator Fluo-8 to detect potential Ca^2+^ activities (transients and spikes) in the differentiating cells, namely VM/mDA NSCs/NPCs (mScarlet-positive) or postmitotic mDA precursors and neurons (mScarlet-negative). However, the use of this novel red fluorescent reporter hiPSC line might be extended to any other application in this regard. Furthermore, our analyses were focused on this type of neural cells and the in vitro generation of human NSCs/NPCs with a VM and mDA identity. Because NESTIN is expressed in several other tissue and stem cell types of the body [14], and because the *NES* promoter, first intron and second intron (harboring a muscle-specific and a CNS-specific enhancer, respectively [34,38]) were left intact in the targeted *NES*-mScarlet #18 hiPSC line, it is reasonable to assume that this novel reporter hiPSC line might also be useful for studies in other stem cell and regenerative medicine fields. This includes, but is not restricted to, the vast area of mesenchymal and hematopoietic stem cells likewise expressing this intermediate filament and implicated in several bone marrow malignancies [14].

## Figures and Tables

**Table 1:** Primers used for genotyping.

Name	Sequence (5′–3′)
P1	GTGGGATGGAGGATGCAGGC
P2	GCTCCTTTGCCACACCCCTT
P3	GAGTTCATGCGGTTCAAGGTGC
P4	CCAGCCCATTGTCTTCTTCTGC
P5	CAGGCCATCTGACCAGGGAAGA
P6	GCACCTTGAACCGCATGAACTC
P7	GGGATTGACTCCAAACTGGAGC
P8	GCAGAAGAAGACAATGGGCTGG

**Table 2:** Marker-gene-specific primer and amplicon sizes for RT-qPCR.

Marker Gene (Stage/Region/Type)	Forward Primer (5′–3′) Reverse Primer (5′–3′)	Tm (°C)	Product Size (bp)
*ACTB* (housekeeping gene)	GCACAGAGCCTCGCCTT	59.68	112
	CCTTGCACATGCCGGAG	58.38	
*ALDH1A1* (SNc DA neurons)	ATCAAAGAAGCTGCCGGGAA	59.96	101
	GCATTGTCCAAGTCGGCATC	59.90	
*BARHL1* (forebrain)	GTACCAGAACCGCAGGACTAAA	60.03	113
	AGAAATAAGGCGACGGGAACAT	60.09	
*CALB1* (VTA DA neurons)	ACGCTGAGCTTTTGCTCACT	60.53	101
	GCAGGTGGGATTCTGCCATT	60.69	
*CCK* (VTA DA neurons)	AAGTGACCGGGACTACATGG	59.10	119
	TGGGTTGGGAGGTTGCTTC	59.85	
*DDC* (mDA neurons)	GACACCATGAACGCAAGTGAAT	59.77	93
	GACCTGGCGTCCCTCAATG	60.45	
*DRD2* (SNc DA neurons)	GCGAGCATCCTGAACTTGTG	59.55	90
	CTTGGAGCTGTAGCGCGTAT	60.25	
*EN1* (VM/mDA NPCs)	CGTGGCTTACTCCCCATTTA	57.30	117
	TCTCGCTGTCTCTCCCTCTC	60.11	
*FOXA2* (VM/mDA NPCs)	CCGTTCTCCATCAACAACCT	57.81	114
	GGGGTAGTGCATCACCTGTT	59.67	
*FOXG1* (forebrain)	TGGCCCATGTCGCCCTTCCT	66.25	77
	GCCGACGTGGTGCCGTTGTA	65.70	
*HOXA2* (hindbrain)	CGTCGCTCGCTGAGTGCCTG	66.07	92
	TGTCGAGTGTGAAAGCGTCGAGG	65.02	
*LMX1A* (mDA NPCs)	CGCATCGTTTCTTCTCCTCT	57.71	150
	CAGACAGACTTGGGGCTCAC	60.32	
*NANOG* (pluripotency)	TGAACCTCAGCTACAAACAG	55.32	138
	TGGTGGTAGGAAGAGTAAAG	53.69	
*NES* (NSCs)	GCGTTGGAACAGAGGTTGGA	60.53	327
	TGGGAGCAAAGATCCAAGAC	57.50	
*NES-mScarlet* (bicistronic mRNA)	GTTCCTGAAGTTCACTCAGAGGG	60.56	72
	CTCGCCCTCGATCTCGAACT	60.44	
*OTX2* (VM/mDA NPCs)	ACAAGTGGCCAATTCACTCC	58.38	62
	GAGGTGGACAAGGGATCTGA	58.43	
*PAX6* (forebrain, neural rosettes)	TAAGGATGTTGAACGGGCAG	58.67	126
	TGGTATTCTCTCCCCCTCCT	57.89	
*PITX3* (mDA neurons)	AGAGGACGGTTCGCTGAAAAA	60.20	93
	TTCCTCTGGAAGGTCGCCTC	61.26	
*POU5F1* (pluripotency)	AGCGAACCAGTATCGAGAAC	57.44	118
	TTACAGAACCACACTCGGAC	57.19	
*mScarlet* (reporter)	GACATCACCTCCCACAACGA	59.68	95
	TTGTACAGCTCGTCCATGCC	60.39	
*SLC6A3 (DAT)* (mDA neurons)	CAAATGCTCCGTGGGACTCA	60.32	109
	CACTCCGTTCTGCTCCTTGA	59.68	
*SOX2* (pluripotency + NSCs)	ATGGGTTCGGTGGTCAAGTC	59.96	426
	GTGGATGGGATTGGTGTTCTC	58.63	
*TH* (mDA neurons)	GGACCTCCACACTGAGCCAT	61.56	107
	ATGATGGCCTCTGCCTGCTT	61.93	
*TUBB3* (neurons)	TTCTCACAAGTACGTGCCTCG	60.34	91
	GAAGAGATGTCCAAAGGCCCC	60.68	

**Table 3:** Primary antibodies for ICC and WB.

Antibody	Species	Dilution	Catalogue Nr.	Manufacturer
β-Actin	Mouse	1:5000	60008	proteintech ^1^
CALB1	Rabbit	1:2000	CB38	Swant
EN1	Mouse	1:100	4GII	DSHB ^2^
LMX1	Rabbit	1:1000	AB10533	Merck Millipore
NANOG	Rabbit	1:500	ab21624	abcam
NESTIN	Rabbit	1:500	ABD69	Merck Millipore
OTX2	Goat	1:2000	AF1979	R&D Systems
OCT4 (POU5F1)	Rabbit	2 µg/mL	ab19857	abcam
RFP	Mouse	1:500	409 011	Synaptic Systems
RFP	Mouse	1:1000	6g6	Chromotek ^1^
SOX2	Rabbit	1:250	ab97959	abcam
TRA-1-60	Mouse	1:500	ab16288	abcam
TH	Mouse	1:1000	MAB318	Merck Millipore
TH	Rabbit	1:1000	AB152	Merck Millipore
TUBB3	Mouse	1:1000	801213	BioLegend

^1^ For WB. ^2^ Developmental Studies Hybridoma Bank, University of Iowa, USA.

**Table 4:** Secondary antibodies for ICC and WB.

Antibody	Dilution	Cat. Nr.
Donkey anti-Mouse IgG Alexa Fluor Plus 488	1:1500	A32766 ^1^
Donkey anti-Mouse IgG Alexa Fluor Plus 555	1:1500	A32773 ^1^
Donkey anti-Rabbit IgG Alexa Fluor Plus 488	1:1500	A32790 ^1^
Donkey anti-Rabbit IgG Alexa Fluor Plus 555	1:1500	A32794^1^
Donkey anti-Goat IgG Alexa Fluor Plus 555	1:1500	A32816 ^1^
Goat anti-Mouse IgG HRPO	1:4000:	SBA-1031-05 ^2^
Goat anti-Rabbit IgG HRPO	1:10000	ab6721 ^3^

^1^ Invitrogen/Thermo Fisher Scientific, for ICC. ^2^ Biozol (Eching, Germany), for WB. ^3^ abcam, for WB.

## Data Availability

Data is contained within the article and Supplementary Material.

## Supplementary Materials

The following are available online at https://www.mdpi.com/article/10.3390/cells11020268/s1, Figure S1: PCR genotyping of *NES*-mScarlet hiPSC clones using primer pairs P5/P6 and P7/8, Figure S2: Karyotyping of *NES*-mScarlet hiPSC clones #7, #18, #21 and #23 using an Illumina platform for SNP analysis, Figure S3: Analysis of off-target sites in *NES*-mScarlet hiPSC clones, Figure S4: *NES*-mScarlet hiPSCs differentiate into genuine VM NSCs/NPCs, Figure S5: *NES*-mScarlet hiPSCs are accurate reporters of human NSCs/NPCs, Figure S6: Live cell imaging of *NES*-mScarlet hiPSC-derived NSCs/NPCs, Figure S7: Gate setting of the flow cytometric analysis of *NES*-mScarlet #18 hiPSC-derived NSCs/NPCs and their *wt* (XM001) counterparts (control) on d11 and d23 of the modified differentiation protocol.

## Appendix A. *NES-mScarlet* Vector Sequence (6151 bp)

CTAAATTGTAAGCGTTAATATTTTGTTAAAATTCGCGTTAAATTTTTGTTAAATCAGCTCA-TTTTTTAACCAATAGGCCGAAATCGGCAAAATCCCTTATAAATCAAAAGAATAGACCGAGATAGGGTTGAGTGGCCGCTACAGGGCGCTCCCATTCGCCATTCAGGCTGCGCAACTGTT-GGGAAGGGCGTTTCGGTGCGGGCCTCTTCGCTATTACGCCAGCTGGCGAAAGGGGGATG-TGCTGCAAGGCGATTAAGTTGGGTAACGCCAGGGTTTTCCCAGTCACGACGTTGTAAAACGACGGCCAGTGAGCGCGACGTAATACGACTCACTATAGGGCGAATTGGCGGAAGGCCGTCAAGGCCTAGGCGCGCCAAAGCTTCCACCCCTGGAAGAGGAGAGTTTGGAGGCAAAGA-GGGTTCAGGGCTTGGAAGGGCCTAGAAAGGACCTAGAGGAGGCAGGTGGTCTGGGGAC-AGAGTTCTCCGAGCTGCCTGGGAAGAGCAGAGACCCTTGGGAGCCTCCCAGGGAGGGTA-GGGAGGAGTCAGAGGCTGAGGCCCCCAGGGGAGCAGAGGAGGCGTTCCCTGCTGAGAC-CCTGGGCCACACTGGAAGTGATGCCCCTTCACCTTGGCCTCTGGGGTCAGAGGAAGCTG-AGGAGGATGTACCACCAGTGCTGGTCTCCCCCAGCCCAACGTACACCCCGATCCTGGAAGATGCCCCTGGGCCTCAGCCTCAGGCTGAAGGGAGTCAGGAGGCTAGCTGGGGGGTGCAGGGGAGGGCTGAAGCCCTGGGGAAAGTAGAGAGCGAGCAGGAGGAGTTGGGTTCTGGGGAGATCCCCGAGGGCCCCCAGGAGGAAGGGGAGGAGAGCAGAGAAGAGAGCGAGGAG-GATGAGCTCGGGGAGACCCTTCCAGACTCCACTCCCCTGGGCTTCTACCTCAGGTCCCCC-ACCTCCCCCAGGTGGGACCCCACTGGAGAGCAGAGGCCACCCCCTCAAGGGGAGACTGGAAAGGAGGGCTGGGATCCTGCTGTCCTGGCTTCCGAGGGCCTTGAGGCCCCACCCTCAGAAAAGGAGGAGGGGGAGGAGGGAGAAGAGGAGTGTGGCCGTGACTCTGACCTGTCAGA-AGAATTTGAGGACCTGGGGACTGAGGCACCTTTTCTTCCTGGGGTCCCTGGGGAGGTGGC-AGAACCTCTGGGCCAGGTGCCCCAGCTGCTACTGGATCCTGCAGCCTGGGATCGAGATGG-GGAGTCCGATGGGTTTGCAGATGAGGAAGAAAGTGGGGAGGAGGGAGAGGAGGATCAGGAGGAGGGGAGGGAGCCAGGGGCTGGGCGGTGGGGGCCAGGGTCTTCTGTTGGCAGCCT-CCAGGCCCTGAGTAGCTCCCAGAGAGGGGAATTCCTGGAGTCTGATTCTGTGAGTGTCAGTGTCCCCTGGGATGACAGCTTGAGGGGTGCAGTGGCTGGTGCCCCCAAGACTGCCCTGGAAACGGAGTCCCAGGACAGTGCTGAGCCTTCTGGCTCAGAGGAAGAGTCTGACCCTGTTTC-CTTGGAGAGGGAGGACAAAGTCCCTGGCCCTCTAGAGATCCCCAGTGGGATGGAGGATG-CAGGCCCAGGGGCAGACATCATTGGTGTTAATGGCCAGGGTCCCAACTTGGAGGGGAAG-TCACAGCATGTGAATGGGGGAGTGATGAACGGGCTGGAGCAGTCTGAGGAAGTGGGGCA-AGGAATGCCGCTAGTCTCTGAGGGAGACCGAGGGAGCCCCTTTCAGGAGGAGGAGGGG-AGTGCTCTGAAGACCTCTTGGGCAGGGGCTCCTGTTCACCTGGGCCAGGGTCAGTTCCTG-AAGTTCACTCAGAGGGAAGGAGATAGAGAGTCCTGGTCCTCAGGGGAGGACAGGGCAA-AGAGGGCCACGAACTTCTCTCTGTTAAAGCAAGCAGGAGATGTTGAAGAAAACCCCGGG-CCTGATATCATGGTGAGCAAGGGCGAGGCAGTGATCAAGGAGTTCATGCGGTTCAAGGT-GCACATGGAGGGCTCCATGAACGGCCACGAGTTCGAGATCGAGGGCGAGGGCGAGGGC-CGCCCCTACGAGGGCACCCAGACCGCCAAGCTGAAGGTGACCAAGGGTGGCCCCCTGCC-CTTCTCCTGGGACATCCTGTCCCCTCAGTTCATGTACGGCTCCAGGGCCTTCACCAAGCAC-CCCGCCGACATCCCCGACTACTATAAGCAGTCCTTCCCCGAGGGCTTCAAGTGGGAGCGC-GTGATGAACTTCGAGGACGGCGGCGCCGTGACCGTGACCCAGGACACCTCCCTGGAGGA-CGGCACCCTGATCTACAAGGTGAAGCTCCGCGGCACCAACTTCCCTCCTGACGGCCCCGTAATGCAGAAGAAGACAATGGGCTGGGAAGCGTCCACCGAGCGGTTGTACCCCGAGGACGGCGTGCTGAAGGGCGACATTAAGATGGCCCTGCGCCTGAAGGACGGCGGCCGCTACCT-GGCGGACTTCAAGACCACCTACAAGGCCAAGAAGCCCGTGCAGATGCCCGGCGCCTACA-ACGTCGACCGCAAGTTGGACATCACCTCCCACAACGAGGACTACACCGTGGTGGAACAGTACGAACGCTCCGAGGGCCGCCACTCCACCGGCGGCATGGACGAGCTGTACAAGTAATG-AGATATCGAAAAGACCATCTGCCCGGCACTGGGGACTTAGGGGTGCGGGGAGGGGAAG-GACGCCTCCAAGCCCGCTCCCTGCTCAGGAGCAGCACTCTTAACTTACGATCTCTTGACA-TATGGTTTCTGGCTGAGAGGCCTGGCCCGCTAAGGTGAAAAGGGGTGTGGCAAAGGAGC-CTACTCCAAGAATGGAGGCTGTAGGAATATAACCTCCCACCCTGCAAAGGGAATCTCTTGCCTGCTCCATCTCATAGGCTAAGTCAGCTGAATCCCGATAGTACTAGGTCCCCTTCCCTCCGCATCCCGTCAGCTGGAAAAGGCCTGTGGCCCAGAGGCTTCTCCAAAGGGAGGGTGAC-ATGCTGGCTTTTGTGCCCAAGCTCACCAGCCCTGCGCCACCTCACTGCAGTAGTGCACCA-TCTCACTGCAGTAGCACGCCCTCCTGGGCCGTCTGGCCTGTGGCTAATGGAGGTGACGGCACTCCCATGTGCTGACTCCCCCCATCCCTGCCACGCTGTGGCCCTGCCTGGCTAGTCCCT-GCCTGAATAAAGTAATGCCTCCGCTTCAAAGTCTGGACTCTTCTTTCCCCCACTCAGAGG-GGACCCTCAGGGTTAGTACAGGAGAGAGATGGCGGGGGCGGGCGGGGGGTGGCGGGGGAGAGGAGGAAGCTGGTGCTGGGGAGGGGCTGCCTCCTGAGGAGAGGAGGACCTGGGG-GGATGAGGGCAGACAGACCCAAGCTGCAGGGAGCAGTGCTGGGGCCTGGGGAGGGCCA-GGGGCTGTTGCTTGGCTGGGGGAGGCCAGTCCAGGTGACCTCAGCGCCTGCAGAAGCCA-GGCCCATCATCATCTCTGCCCACAGTACTGATTACAGGGATTAGGCCTTGGGGCCACCCC-CACCCTTGTATACCCCCCCCCATTAGTGCCTTGGTGCCGCCAGCATAATAAGCCCCCTTGT-CTCTAAACCCAAACAAGTTGTCTGGCCTGATCTCCTCAGCCTGACTGGGTGGGAGCAGGG-TGAGGGAAGAGGCAGGCCTGTTCTTCCTCAGTCTGGCTGCATGGCCTCAGGTCAGCATTG-ATCCTCTCTGAGCCTCAGACTCTGCCACACAATGAGAGAGGCTGGGCCAGGTGACCTGGGAGCCTCCCAGGGCCCAGGAGAGCAGGAAGGAGGCTGAAGACAGCGTAGCTCTTCAGTAGGACTTCCTTCACACGCTGCAGCAGGAGGCAGGGAAGAAGGGAAAGTGGAGGGGGAGC-TGAACTGTCAGGGTGGGAGCTAGGGGTGTGCTGATGACAGTGCTGTGCTGGCAGCAGCTT-AGCCCGAGGGTCCTCCAGCCACAGTGTCTCCTCCTCACCCCACTTCCCAGGAGCTCTCTGT-GTCCCTGTGTAGGACAGGGAGGGGACCAGGGGTTCCTGGATTAGGTGGGGCCTGCAGGC-CTTGAAGTCAGGATACCCAAATAGAAACCACTCGCTGCTTCAGCTCTGCGGGGAAGGAAGATCTGGCGACACTTCTAAATAGAAGCTTATTAATTAACTGGCCTCATGGGCCTTCCGCTCACTGCCCGCTTTCCAGTCGGGAAACCTGTCGTGCCAGCTGCATTAACATGGTCATAGCTG-TTTCCTTGCGTATTGGGCGCTCTCCGCTTCCTCGCTCACTGACTCGCTGCGCTCGGTCGTTC-GGGTAAAGCCTGGGGTGCCTAATGAGCAAAAGGCCAGCAAAAGGCCAGGAACCGTAAA-AAGGCCGCGTTGCTGGCGTTTTTCCATAGGCTCCGCCCCCCTGACGAGCATCACAAAAAT-CGACGCTCAAGTCAGAGGTGGCGAAACCCGACAGGACTATAAAGATACCAGGCGTTTCC-CCCTGGAAGCTCCCTCGTGCGCTCTCCTGTTCCGACCCTGCCGCTTACCGGATACCTGTCC-GCCTTTCTCCCTTCGGGAAGCGTGGCGCTTTCTCATAGCTCACGCTGTAGGTATCTCAGTT-CGGTGTAGGTCGTTCGCTCCAAGCTGGGCTGTGTGCACGAACCCCCCGTTCAGCCCGACCGCTGCGCCTTATCCGGTAACTATCGTCTTGAGTCCAACCCGGTAAGACACGACTTATCGC-CACTGGCAGCAGCCACTGGTAACAGGATTAGCAGAGCGAGGTATGTAGGCGGTGCTAC-AGAGTTCTTGAAGTGGTGGCCTAACTACGGCTACACTAGAAGAACAGTATTTGGTATCTG-CGCTCTGCTGAAGCCAGTTACCTTCGGAAAAAGAGTTGGTAGCTCTTGATCCGGCAAACA-AACCACCGCTGGTAGCGGTGGTTTTTTTGTTTGCAAGCAGCAGATTACGCGCAGAAAAAA-AGGATCTCAAGAAGATCCTTTGATCTTTTCTACGGGGTCTGACGCTCAGTGGAACGAAAA-CTCACGTTAAGGGATTTTGGTCATGAGATTATCAAAAAGGATCTTCACCTAGATCCTTTTA-AATTAAAAATGAAGTTTTAAATCAATCTAAAGTATATATGAGTAAACTTGGTCTGACAGT-TACCAATGCTTAATCAGTGAGGCACCTATCTCAGCGATCTGTCTATTTCGTTCATCCATAG-TTGCCTGACTCCCCGTCGTGTAGATAACTACGATACGGGAGGGCTTACCATCTGGCCCCA-GTGCTGCAATGATACCGCGAGAACCACGCTCACCGGCTCCAGATTTATCAGCAATAAACCAGCCAGCCGGAAGGGCCGAGCGCAGAAGTGGTCCTGCAACTTTATCCGCCTCCATCCAGTCTATTAATTGTTGCCGGGAAGCTAGAGTAAGTAGTTCGCCAGTTAATAGTTTGCGCAACGTTGTTGCCATTGCTACAGGCATCGTGGTGTCACGCTCGTCGTTTGGTATGGCTTCATTCAGCTCCGGTTCCCAACGATCAAGGCGAGTTACATGATCCCCCATGTTGTGCAAAAAAGCGGT-TAGCTCCTTCGGTCCTCCGATCGTTGTCAGAAGTAAGTTGGCCGCAGTGTTATCACTCATG-GTTATGGCAGCACTGCATAATTCTCTTACTGTCATGCCATCCGTAAGATGCTTTTCTGTGA-CTGGTGAGTACTCAACCAAGTCATTCTGAGAATAGTGTATGCGGCGACCGAGTTGCTCTTGCCCGGCGTCAATACGGGATAATACCGCGCCACATAGCAGAACTTTAAAAGTGCTCATCATTGGAAAACGTTCTTCGGGGCGAAAACTCTCAAGGATCTTACCGCTGTTGAGATCCAGTT-CGATGTAACCCACTCGTGCACCCAACTGATCTTCAGCATCTTTTACTTTCACCAGCGTTTC-TGGGTGAGCAAAAACAGGAAGGCAAAATGCCGCAAAAAAGGGAATAAGGGCGACACG-GAAATGTTGAATACTCATACTCTTCCTTTTTCAATATTATTGAAGCATTTATCAGGGTTATT-GTCTCATGAGCGGATACATATTTGAATGTATTTAGAAAAATAAACAAATAGGGGTTCCGC-GCACATTTCCCCGAAAAGTGCCAC

## Appendix B

**Table A1:** Composition of all media used for hiPSC culture and differentiation in this work.

Medium Name	Added Factors	Ingredients	Final Concentration
hiPSC-medium		StemMACS™ iPS-Brew medium	1x
N2 medium		DMEM:F12 and Neurobasal	1:1 mixture
		N-2 supplement	1% vol/vol
		GlutaMAX	1% vol/vol (2 mM)
		Penicillin/streptomycin	0.2% vol/vol
	+d0–d9:	SB431542 (TGFβ inhibitor)	10 µM
	+d0–d10:	Recombinant human Noggin (BMP inhibitor)	100 ng/mL
		Recombinant human SHH-C24II	375 ng/mL
	+d2–d10:	Recombinant human FGF8b	100 ng/mL
	+d3–d10:	CHIR99021 (GSK3β inhibitor)	0.6 µM
Freezing medium		DMEM:F12 and Neurobasal	1:1 mixture
		N-2 supplement	1% vol/vol
		B-27 supplement without vitamin A	4% vol/vol
		GlutaMAX	1% vol/vol (2 mM)
		DMSO	10% vol/vol
B27 medium		Neurobasal	
		B-27 supplement without vitamin A	2% vol/vol
		GlutaMAX	1% vol/vol (2 mM)
		Penicillin/streptomycin	0.2% vol/vol
	+d10–d16:	Recombinant human FGF8b	100 ng/mL
		CHIR99021 (GSK3β inhibitor)	0.6 µM
	+d10–remaining culture time	Recombinant human BDNF	20 ng/mL
		L-Ascorbic acid	0.2 mM
	+d16–remaining culture time	Recombinant human GDNF	10 ng/mL
		Dibutyryl-cyclic AMP (db-cAMP)	500 µM
		N-[(3,5-difluorophenyl)acetyl]-l-alanyl-2-phenyl]glycine-1, 1-dimethylethyl ester (DAPT)	1 µM

## References

[1] Kelava I., Lancaster M.A. (2016). Stem Cell Models of Human Brain Development. Cell Stem Cell.

[2] Kim J., Koo B.-K., Knoblich J.A. (2020). Human organoids: Model systems for human biology and medicine. Nat. Rev. Mol. Cell Biol..

[3] Rowe R.G., Daley G.Q. (2019). Induced pluripotent stem cells in disease modelling and drug discovery. Nat. Rev. Genet..

[4] Sterneckert J.L., Reinhardt P., Scholer H.R. (2014). Investigating human disease using stem cell models. Nat. Rev. Genet..

[5] Goldman S.A. (2016). Stem and Progenitor Cell-Based Therapy of the Central Nervous System: Hopes, Hype, and Wishful Thinking. Cell Stem Cell.

[6] Parmar M., Grealish S., Henchcliffe C. (2020). The future of stem cell therapies for Parkinson disease. Nat. Rev. Neurosci..

[7] Balestrino R., Schapira A.H.V. (2020). Parkinson disease. Eur. J. Neurol..

[8] Kim T.W., Koo S.Y., Studer L. (2020). Pluripotent Stem Cell Therapies for Parkinson Disease: Present Challenges and Future Opportunities. Front. Cell Dev. Biol..

[9] Parmar M. (2018). Towards stem cell based therapies for Parkinson’s disease. Development.

[10] Poulin J.-F., Gaertner Z., Moreno-Ramos O.A., Awatramani R. (2020). Classification of Midbrain Dopamine Neurons Using Single-Cell Gene Expression Profiling Approaches. Trends Neurosci..

[11] Blaess S., Ang S.-L. (2015). Genetic control of midbrain dopaminergic neuron development. Wiley Interdiscip. Rev. Dev. Biol..

[12] La Manno G., Gyllborg D., Codeluppi S., Nishimura K., Salto C., Zeisel A., Borm L.E., Stott S.R.W., Toledo E.M., Villaescusa J.C. (2016). Molecular Diversity of Midbrain Development in Mouse, Human, and Stem Cells. Cell.

[13] Nolbrant S., Heuer A., Parmar M., Kirkeby A. (2017). Generation of high-purity human ventral midbrain dopaminergic progenitors for in vitro maturation and intracerebral transplantation. Nat. Protoc..

[14] Bernal A., Arranz L. (2018). Nestin-expressing progenitor cells: Function, identity and therapeutic implications. Cell. Mol. Life Sci..

[15] Kim K., Higashi M., Fumino S., Tajiri T. (2019). Derivation of neural stem cells from human teratomas. Stem Cell Res..

[16] Eze U.C., Bhaduri A., Haeussler M., Nowakowski T.J., Kriegstein A.R. (2021). Single-cell atlas of early human brain development highlights heterogeneity of human neuroepithelial cells and early radial glia. Nat. Neurosci..

[17] Lee Y., Choi H.Y., Kwon A., Park H., Park M., Kim Y.-O., Kwak S., Koo S.K. (2019). Generation of a NESTIN-EGFP reporter human induced pluripotent stem cell line, KSCBi005-A-1, using CRISPR/Cas9 nuclease. Stem Cell Res..

[18] Noisa P., Urrutikoetxea-Uriguen A., Li M., Cui W. (2010). Generation of human embryonic stem cell reporter lines expressing GFP specifically in neural progenitors. Stem Cell Rev. Rep..

[19] Wang X., Sterr M., Burtscher I., Chen S., Hieronimus A., Machicao F., Staiger H., Häring H.-U., Lederer G., Meitinger T. (2018). Genome-wide analysis of PDX1 target genes in human pancreatic progenitors. Mol. Metab..

[20] Yumlu S., Bashir S., Stumm J., Kühn R. (2019). Efficient Gene Editing of Human Induced Pluripotent Stem Cells Using CRISPR/Cas9. Methods Mol. Biol..

[21] Li X.-L., Li G.-H., Fu J., Fu Y.-W., Zhang L., Chen W., Arakaki C., Zhang J.-P., Wen W., Zhao M. (2018). Highly efficient genome editing via CRISPR-Cas9 in human pluripotent stem cells is achieved by transient BCL-XL overexpression. Nucleic Acids Res..

[22] Fang J., Qian J.-J., Yi S., Harding T.C., Tu G.H., VanRoey M., Jooss K. (2005). Stable antibody expression at therapeutic levels using the 2A peptide. Nat. Biotechnol..

[23] Bindels D.S., Haarbosch L., van Weeren L., Postma M., Wiese K.E., Mastop M., Aumonier S., Gotthard G., Royant A., Hink M.A. (2017). mScarlet: A bright monomeric red fluorescent protein for cellular imaging. Nat. Methods.

[24] Haeussler M., Schönig K., Eckert H., Eschstruth A., Mianné J., Renaud J.-B., Schneider-Maunoury S., Shkumatava A., Teboul L., Kent J. (2016). Evaluation of off-target and on-target scoring algorithms and integration into the guide RNA selection tool CRISPOR. Genome Biol..

[25] de Rus Jacquet A. (2019). Preparation and Co-Culture of iPSC-Derived Dopaminergic Neurons and Astrocytes. Curr. Protoc. Cell Biol..

[26] Kriks S., Shim J.W., Piao J., Ganat Y.M., Wakeman D.R., Xie Z., Carrillo-Reid L., Auyeung G., Antonacci C., Buch A. (2011). Dopamine neurons derived from human ES cells efficiently engraft in animal models of Parkinson’s disease. Nature.

[27] Schmittgen T.D., Livak K.J. (2008). Analyzing real-time PCR data by the comparative C(T) method. Nat. Protoc..

[28] Anderson N.C., Chen P.-F., Meganathan K., Afshar Saber W., Petersen A.J., Bhattacharyya A., Kroll K.L., Sahin M. (2021). Balancing serendipity and reproducibility: Pluripotent stem cells as experimental systems for intellectual and developmental disorders. Stem Cell Rep..

[29] Kim T.W., Piao J., Koo S.Y., Kriks S., Chung S.Y., Betel D., Socci N.D., Choi S.J., Zabierowski S., Dubose B.N. (2021). Biphasic Activation of WNT Signaling Facilitates the Derivation of Midbrain Dopamine Neurons from hESCs for Translational Use. Cell Stem Cell.

[30] Fischer T., Faus-Kessler T., Welzl G., Simeone A., Wurst W., Prakash N. (2011). Fgf15-mediated control of neurogenic and proneural gene expression regulates dorsal midbrain neurogenesis. Dev. Biol..

[31] Carola G., Malagarriga D., Calatayud C., Pons-Espinal M., Blasco-Agell L., Richaud-Patin Y., Fernandez-Carasa I., Baruffi V., Beltramone S., Molina E. (2021). Parkinson’s disease patient-specific neuronal networks carrying the LRRK2 G2019S mutation unveil early functional alterations that predate neurodegeneration. NPJ Parkinsons Dis..

[32] Rienecker K.D.A., Poston R.G., Saha R.N. (2020). Merits and Limitations of Studying Neuronal Depolarization-Dependent Processes Using Elevated External Potassium. ASN Neuro.

[33] Heidenreich M., Zhang F. (2016). Applications of CRISPR-Cas systems in neuroscience. Nat. Rev. Neurosci..

[34] Lothian C., Prakash N., Lendahl U., Wahlström G.M. (1999). Identification of both general and region-specific embryonic CNS enhancer elements in the nestin promoter. Exp. Cell Res..

[35] Li W., Chen S., Li J.-Y. (2015). Human induced pluripotent stem cells in Parkinson’s disease: A novel cell source of cell therapy and disease modeling. Prog. Neurobiol..

[36] Oosterveen T., Garção P., Moles-Garcia E., Soleilhavoup C., Travaglio M., Sheraz S., Peltrini R., Patrick K., Labas V., Combes-Soia L. (2021). Pluripotent stem cell derived dopaminergic subpopulations model the selective neuron degeneration in Parkinson’s disease. Stem Cell Rep..

[37] Nouri P., Götz S., Rauser B., Irmler M., Peng C., Trümbach D., Kempny C., Lechermeier C.G., Bryniok A., Dlugos A. (2020). Dose-Dependent and Subset-Specific Regulation of Midbrain Dopaminergic Neuron Differentiation by LEF1-Mediated WNT1/b-Catenin Signaling. Front. Cell Dev. Biol..

[38] Zimmerman L., Lendahl U., Cunningham M., McKay R., Parr B., Gavin B., Mann J., Vassileva G., McMahon A. (1994). Independent regulatory elements in the nestin gene direct transgene expression to neural stem cells or muscle precursors. Neuron.

