# Supplementary material for: Generation of a NES-mScarlet Red Fluorescent Reporter Human iPSC Line for Live Cell Imaging and Flow Cytometric Analysis and Sorting Using CRISPR-Cas9-Mediated Gene Editing
Source: Cells. 2022 Jan 13;11(2):268. doi: 10.3390/cells11020268 (PMC8773741; doi:10.3390/cells11020268)
Supplement: Supplementary file 1 [file cells-11-00268-s001.zip › Suppl. Figs .pdf]

Generation of a *NES*-mScarlet red fluorescent reporter human iPSC line for live cell imaging and flow cytometric analysis and sorting using CRISPR-Cas9 mediated gene editing

Parivash Nouri, Anja Zimmer, Stefanie Brüggemann, Robin Friedrich, Ralf Kühn and Nilima Prakash

Supplementary Figures:

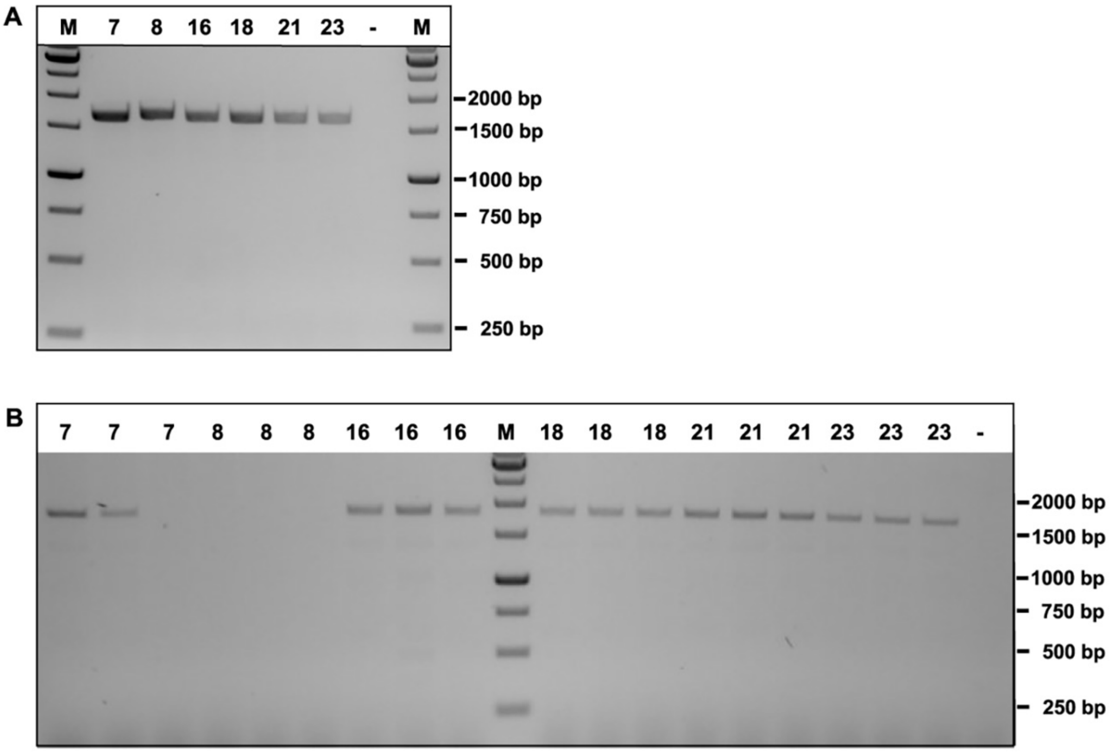

Figure S1. PCR genotyping of *NES*-mScarlet hiPSC clones using primer pairs P5/P6 and P7/8. (**A**) PCR amplification of genomic DNA from XM001 hiPSC clone #7, #8, #16, #18, #21 and #23 using primers P5/P6 (Figure 1) showed the 1659 bp band predicted from the upstream region of the targeted *NES* locus. (**B)** 5 of these clones (except for clone #8) exhibited the predicted 1855 bp band covering the downstream region of the targeted *NES* locus using primers P7/P8 (Figure 1). −, water control; M, size marker.

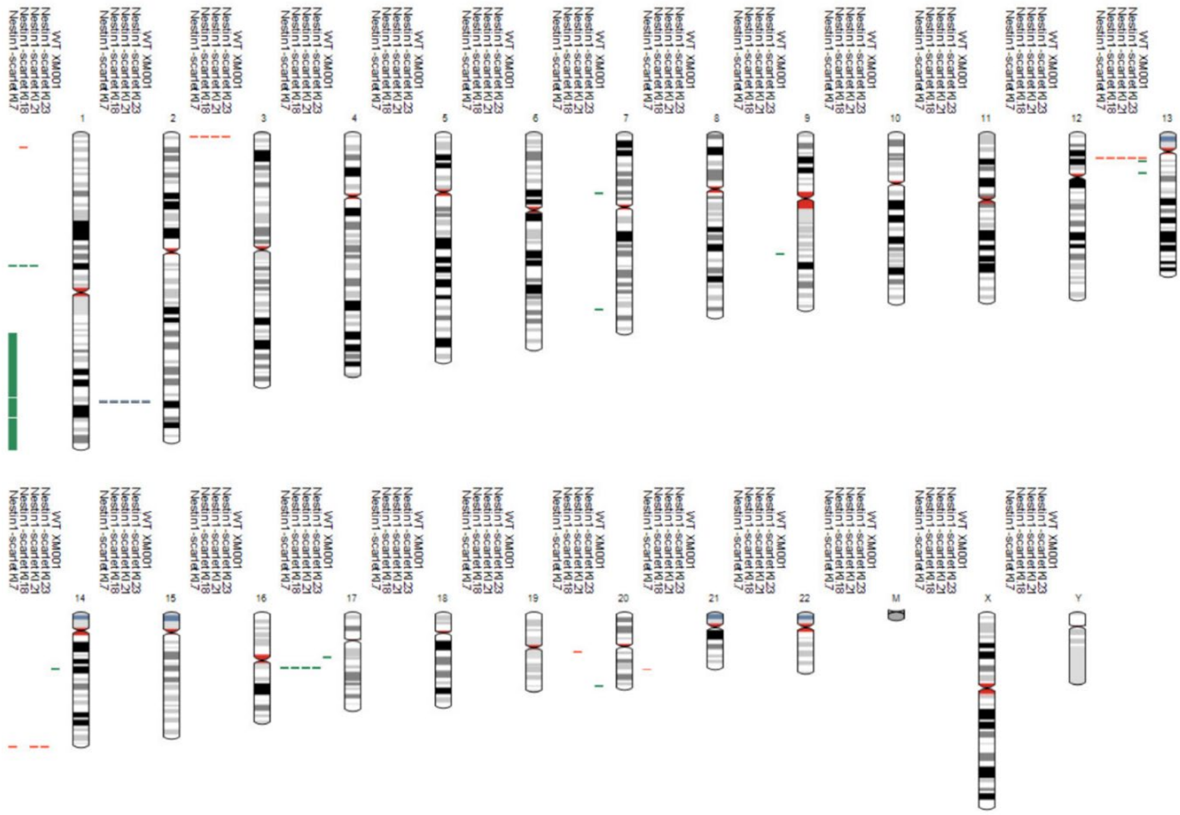

Figure S2. Karyotyping of *NES*-mScarlet hiPSC clones #7, #18, #21 and #23 using an Illumina platform for SNP analysis. Clones #18, #21 and #23 exhibited normal karyotypes, whereas clone #7 showed a duplication on chromosome 1 (green label on left side) and was excluded from further analysis.

A

ON: CTGGTCCTCAGGGGAGGACT AGG	Chrom.	start	end	locus	Primer-for	Primer-rev
OT1:T------------------- G-A	chr1	27074270	27074292	intergenic:FKSG62-SLC9A1	GCACTGCAGCTAGAGGATTCATT	GGTCAAGGCTGCAGTTAGCCATG
OT2:--A------------A---- ---	chr11	100001833	100001855	intron:CNTN5	ACCTCATTCATGGCAATGATCACT	ACTGGGGAAATGACACCTAGCAA
OT3:-----A--G----------- CA-	chr9	120858353	120858375	intron:PHF19	ATGTATGCCTTGGCTGCCAGCT	CAGGGACTACATAACAGGCACC
OT4:----G-T------------- CA-	chr3	51536722	51536744	intergenic:VPRBP-RAD54L2	CTATAAATAAATGCCTGGCACTGG	GAGATTGCAGTGAGCTGAGATTG
OT5:-A-----------------C T--	chr5	150130718	150130740	intron:PDGFRB	GGTGGTGACCTGGCCTCCTA	AGTCTACAGCCCTCCTCCCCC
OT6:--A--------------C-- GA-	chr11	69079096	69079118	intron:TPCN2	CTGCGTCTTCATTGTGTACTACC	GATGCAGTAGAAACCCAGCCTC

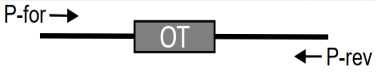

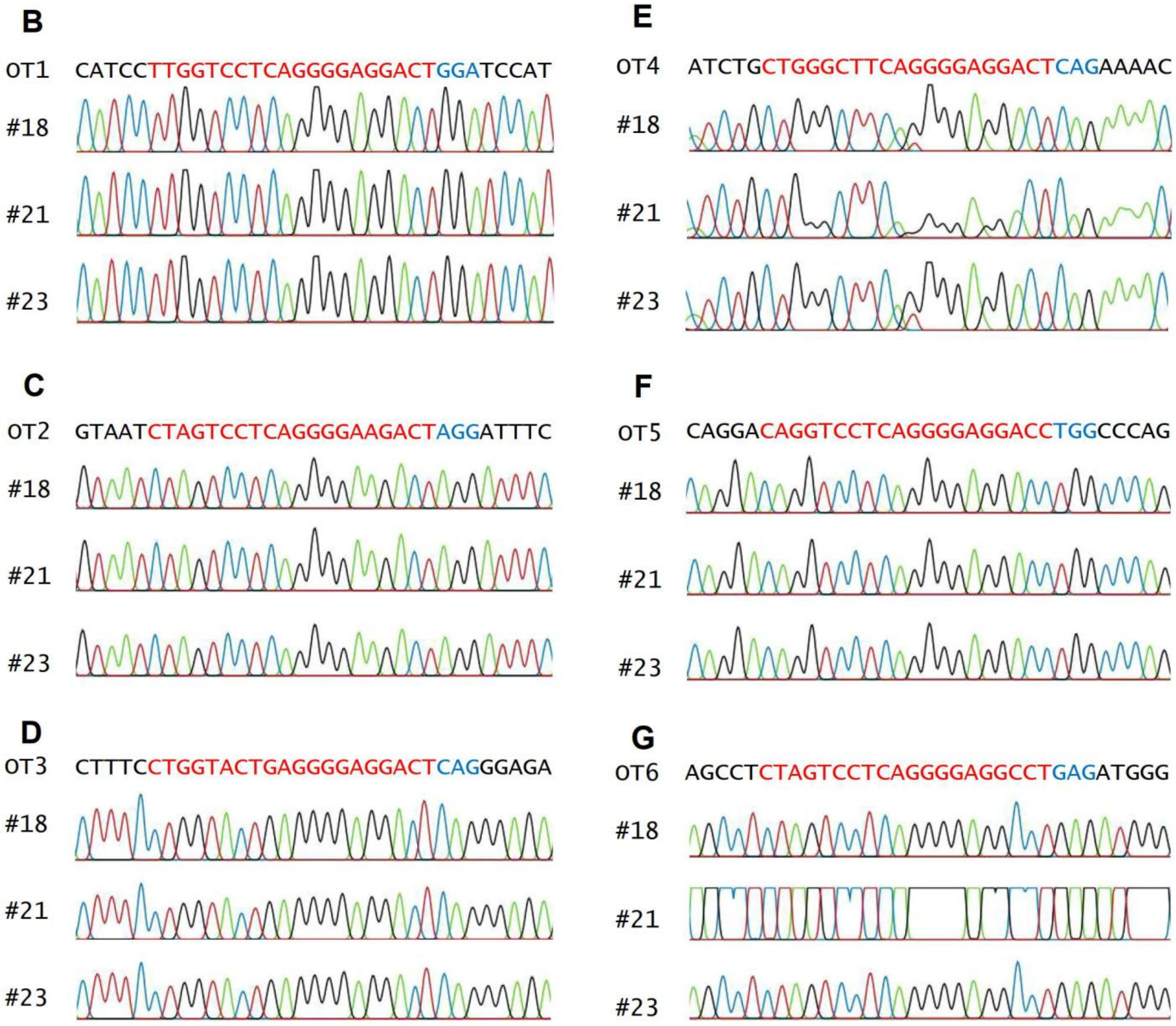

Figure S3. Analysis of off-target sites in *NES*-mScarlet hiPSC clones. (**A**) Genomic regions of predicted top off-target sites OT1-6 as compared to the on-target site were amplified from genomic DNA of *NES*-mScarlet hiPSC clones #18, #21 and #23 using flanking primer pairs (P-for, P-rev). (**B-G**) Sanger sequencing peaks of PCR products OT1 - OT6 from *NES*-mScarlet hiPSC clones in comparison to the genomic sequences. The predicted off-target sequences are shown in red, the PAM site in blue letters. The presence of completely matching wildtype sequences derived from the hiPSC clones verifies the absence of Indel mutations at these sites.

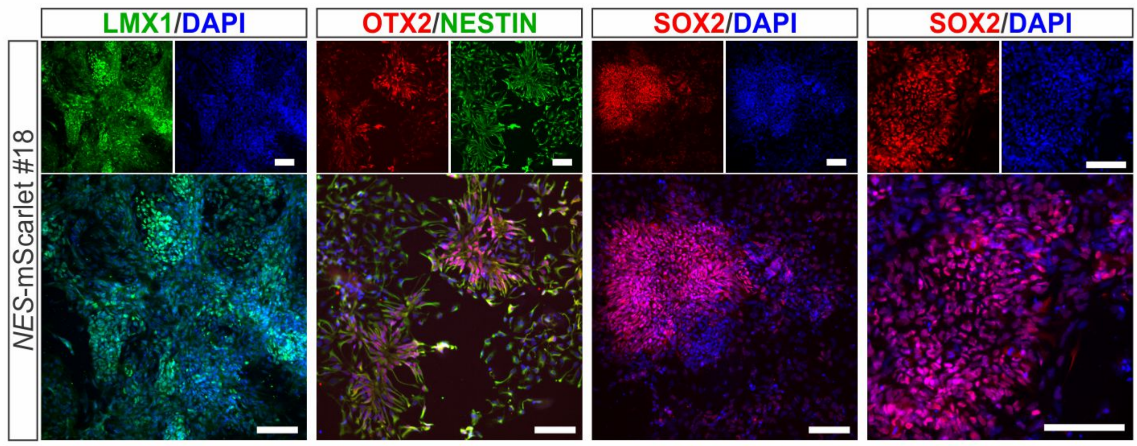

Figure S4. *NES*-mScarlet hiPSCs differentiate into genuine VM NSCs/NPCs. (**a**) ICC for the detection of the VM NSC/NPC marker proteins LMX1, OTX2 and SOX2 in the *NES*-mScarlet #18 hiPSC-derived cells on d31 of the modified differentiation protocol. Top panels are the single green (LMX1 and NESTIN), red (OTX2 and SOX2) and blue (DAPI) channel views of the merged images below. Scale bars: 100 µm.

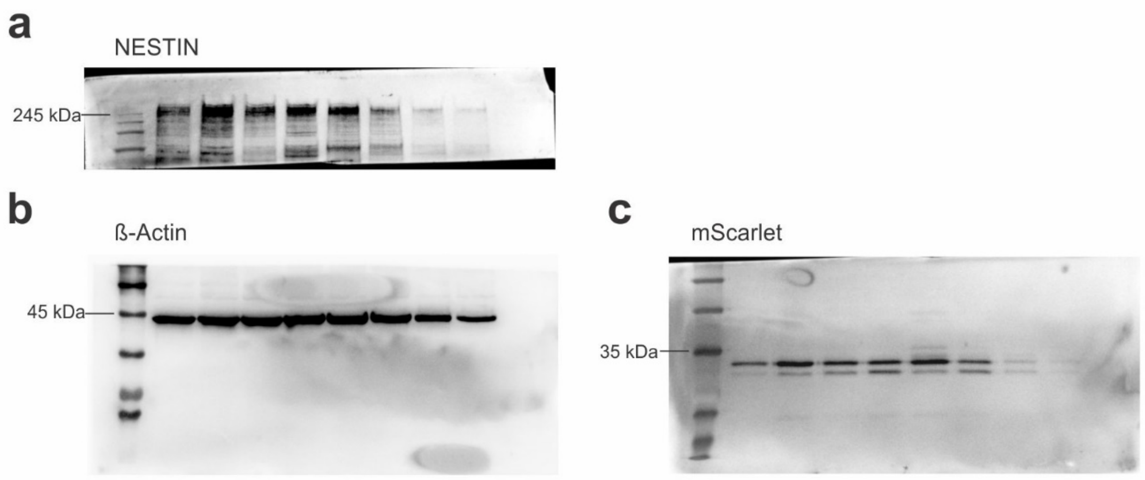

Figure S5. *NES*-mScarlet hiPSCs are accurate reporters of human NSCs/NPCs. Complete views of the Western blots shown in Figure 6 for the detection of full-length human NESTIN (~240 kDa) (**a**), β-Actin (42 kDa) (**b**), and mScarlet (~30 kDa) (**c**) proteins in cell lysates from differentiating *NES*-mScarlet #18 hiPSCs on d7, d11, d16, d23, d31, d41, d51 and d61 (lanes from left to right) of the modified differentiation protocol. Beta-Actin was used as a loading control.

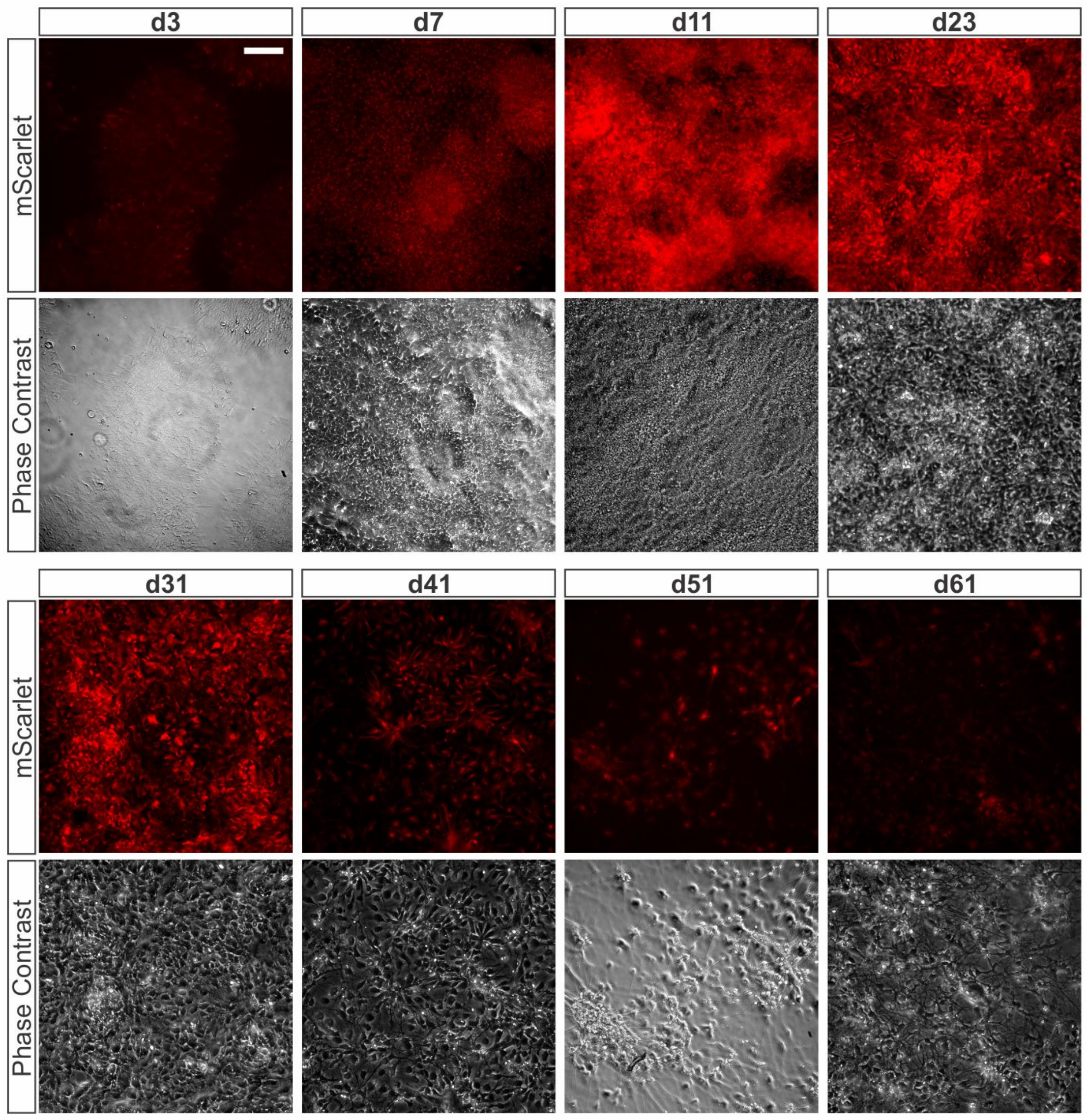

Figure S6. Live cell imaging of *NES*-mScarlet hiPSC-derived NSCs/NPCs. Detection of the red mScarlet fluorescence in *NES*-mScarlet #18 hiPSC-derived cells on d3, d7, d11, d23, d31, d41, d51 and d61 of the modified differentiation protocol. The corresponding phase-contrast images are shown below. Scale bar: 100 μm.

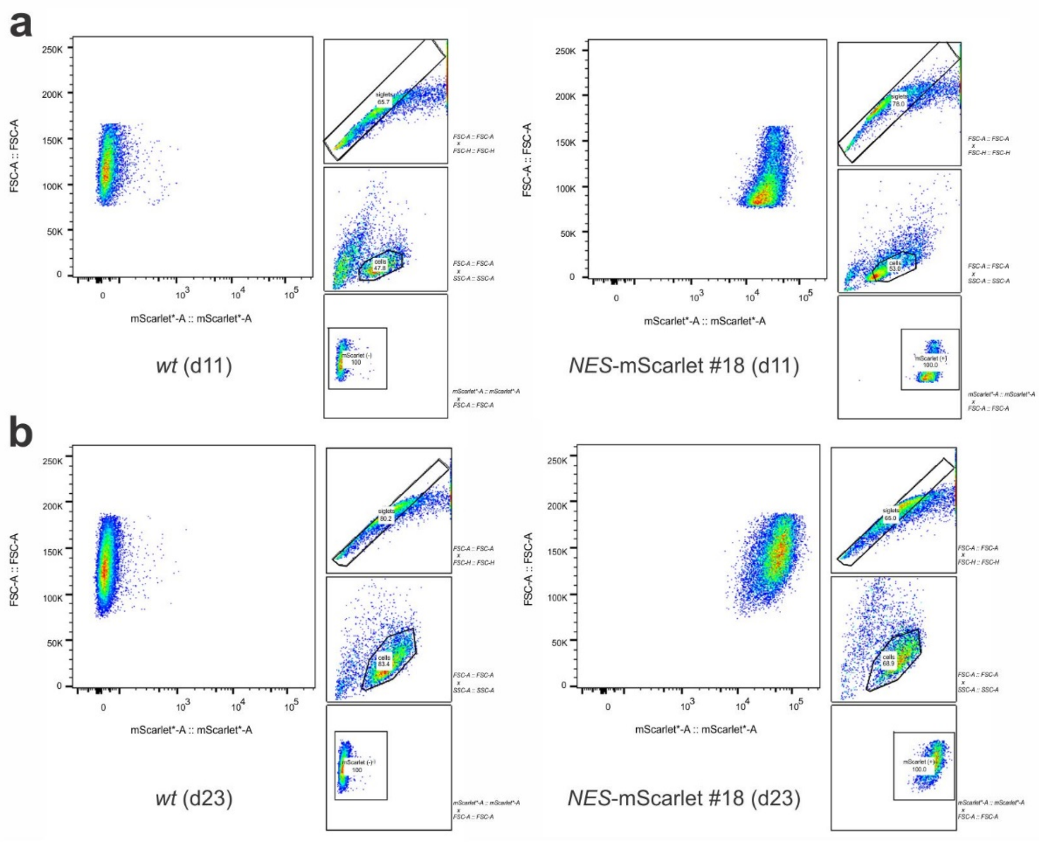

Figure S7. Gate setting of the flow cytometric analysis of *NES*-mScarlet #18 hiPSC-derived NSCs/NPCs and their *wt* (XM001) counterparts (control) on d11 and d23 of the modified differentiation protocol. Scatter plots showing the sequential purification for singlets (gate 1), live (gate 2) and mScarlet⁺ (gate 3) cells.
